# Hybridization and introgression events in cooccurring populations of closely related grasses (Poaceae: *Stipa*) in high mountain steppes of Central Asia

**DOI:** 10.1371/journal.pone.0298760

**Published:** 2024-02-27

**Authors:** Patar Sinaga, Ewelina Klichowska, Arkadiusz Nowak, Marcin Nobis

**Affiliations:** 1 Institute of Botany, Faculty of Biology, Jagiellonian University, Kraków, Poland; 2 Doctoral School of Exact and Natural Sciences, Jagiellonian University, Kraków, Poland; 3 Polish Academy of Sciences Botanical Garden – Center for Biological Diversity Conservation in Powsin, Warszawa, Poland; 4 Botanical Garden of the Wrocław University, Wrocław, Poland; Institute for Biological Research, University of Belgrade, SERBIA

## Abstract

*Stipa* is a genus comprising ca. 150 species found in warm temperate regions of the Old World and around 30% of its representatives are of hybrid origin. In this study, using integrative taxonomy approach, we tested the hypothesis that hybridization and introgression are the explanations of the morphological intermediacy in species belonging to *Stipa* sect. *Smirnovia*, one of the species-rich sections in the mountains of Central Asia. Two novel nothospecies, *S*. *magnifica* × *S*. *caucasica* subsp. *nikolai* and *S*. *lingua* × *S*. *caucasica* subsp. *nikolai*, were identified based on a combination of morphological characters and SNPs markers. SNPs marker revealed that all *S*. *lingua* × *S*. *caucasica* samples were F1 hybrids, whereas most of *S*. *magnifica* × *S*. *caucasica* samples were backcross hybrids. Furthermore, the above mentioned hybrids exhibit transgressive morphological characters to each of their parental species. These findings have implications for understanding the process of hybridization in the genus *Stipa*, particularly in the sect. *Smirnovia*. As a taxonomic conclusion, we describe the two new nothospecies *S*. *× muksuensis* (from Tajikistan) and *S*. *× ochyrae* (from Kyrgyzstan) and present an identification key to species morphologically similar to the taxa mentioned above.

## Introduction

Hybrids occur in approximately 40% of plant families and 16% of genera and there is a strong correlation between phylogeny levels and the possibility of hybridization, implying that some plant groups are more likely to hybridize than others [1]. This process generates speciation and taxonomic divergence as well as increases genetic diversity and plant adaptation, and so contributes to the emergence of angiosperm diversity [2–5]. However, hybridization can also cause a decrease in fitness and genetic swamping [6–8], which breaks species identity and could cause extinction [9]. Due to reduced pollen viability, one common consequence of hybridization is a decrease in fertility in the F1 generation [10–14]. Hybrids may exhibit lower fitness due to various factors such as natural selection against hybrids, hybridization load, and hybrid incompatibilities. The fitness of hybrids is influenced by a variety of factors and processes, therefore the evolutionary consequences of hybridization can vary greatly depending on the cases. Moreover, the simultaneous influence of these factors makes the evolution of hybrids a dynamic process [15].

Hybridization often leads to introgression, where genetic material is transferred from one species to another as a result of backcrossing [16, 17]. Hybrids produced through hybridization have the capacity to backcross repeatedly with either one or both of the progenitors [10, 18]. In addition to mutations and neutral genetic variation, this introgression is one of the processes for the generation of novel alleles [19], which is crucial for environmental adaptation. Numerous studies have demonstrated that new genetic variation obtained through introgression plays a crucial role in enabling adaptation to new environments and facilitating range shifts [19, 20]. Therefore, understanding the prevalence and effects of hybridization in natural populations is critical to gaining insight into the adaptive capacity of hybrids [21–23].

Poaceae is the fifth most diverse family of Angiosperms and the second most diverse monocotyledon family in the world [24]. Due to many examples of hybrids that occur in Poaceae, including intergeneric hybrids such as the wheat-grass cross of *Triticum* × *Agropyron* [25, 26], *Oryzopsis* × *Stipa* [27], *Dendrocalamus* × *Gigantochloa* [28], and intrageneric hybrids such as in *Elymus* [7, 29, 30], *Andropogon* [31], *Elytrigia* [32], *Arundinaria* [33], *Poa* [34] and *Austrostipa* [35], therefore the family constitute an excellent example for research on the role of interspecific hybridization [36–39]. Along with the previously mentioned examples, *Stipa* is one of the genera with over 150 species found in warm temperate regions of Europe, Asia, and North Africa [40–42], with approximately 30% of hybrid origin taxa [40]. The majority of hybrid identifications in *Stipa* (e.g. *S*. × *brozhiana* M. Nobis, *S*. × *gegarkunii* P.A. Smirn., *S*. × *fallax* M. Nobis & A. Nowak, *S*. × *czerepanovii* Kotuch., *S*. *× balkanabatica* M. Nobis & P.D. Gudkova) [40, 43–50] were detected using morphological traits, where most of them, as F1 hybrids, expressed intermediate characters in relation to their parental species. The hybrid origin taxa, however, may express transgressive characters that can be seen as novel traits separating them from their parents and assist in the process of colonization in new environments [51, 52]. Additionally, *Stipa* hybrids may produce viable pollen grains, which make them able to backcross with parental taxa [10, 46, 53]. In addition to phenotypic characters, DNA polymorphism offers various advantages in hybrid detection because it is relatively simple to be examined, has a large number across the genome, and is located in non-coding areas [52]. Furthermore, advances in sequencing technology have made it much easier for researchers to perform studies on a larger scale, faster, and with non-model species. By using a combined approach of morphological characters and DArT sequencing method [54, 55], the hybrid origin of *Stipa* nothospecies were confirmed in: *S*. × *lazkovii* M. Nobis & A. Nowak (F1 hybrid between *S*. *krylovii* Roshev. and *S*. *bungeana* Trin.) [6], *S*. × *heptapotamica* Golosk. (*S*. *lessingiana* Trin. & Rupr. × *S*. *richteriana* Kar. & Kir.) [10], *S*. × *smelanskyi* P.D. Gudkova & M. Nobis (*S*. *richteriana* × *S*. *drobovii* (Tzvel.) Czerep.) [56], an extensive hybridization between *S*. *baicalensis* Roshev., *S*. *capillata* L., *S*. *grandis* P.A. Smirn. and *S*. *krylovii* [53] and hybridization between *S*. *gracilis* Roshev. *× S*. *zeravshanica* M. Nobis [57].

During field studies conducted in high mountain steppes of the Pamir-Alai and Tian Shan Mountains on localities with sympatric populations, we discovered specimens that exhibit intermediate morphological characteristics between closely related taxa of feathergrasses (*Stipa* sect. *Smirnovia* [48, 58–60]). Having in mind that in cooccurring populations, gene flow may impact on higher morphological variability, some specimens were difficult to identify. Such overestimated variability makes the border between closely related taxa almost impossible to determine. Because no studies have been conducted to test the importance and impact of hybridization in cooccurring populations of *Stipa* sect. *Smirnovia* representatives, we aim to: i) provide morphological and molecular evidence of hybridization between closely related species of feathergrasses in sympatric populations of species belonging to sect. *Smirnovia*, ii) demonstrate transgressive characters of the putative hybrids in relation to the characters of their parental taxa, and iii) identify the genetic structure of the population and detect introgression events within examined specimens.

## Materials and methods

### Plant material

Morphological identification was based on specimens preserved in the KRA herbarium (Herbarium of the Jagiellonian University in Kraków). All taxa used in this study represent *Stipa* sect. *Smirnovia* and are characterized by unigeniculate and pilose throughout awns [59]. In total, 501 specimens from *Stipa* were examined in this study, of which eight belong to *S*. *aktauensis* Roshev., 22 to *S*. *magnifica* A. Junge, 21 to *S*. *lingua* Roshev., 29 to *S*. *narynica* M. Nobis, 38 to *S*. *caucasica* Schmalh. subsp. *caucasica*, 120 to *S*. *caucasica* subsp. *nikolai* M. Nobis, A. Nobis & A. Nowak, 131 of *S*. *drobovii* var. *drobovii*, 71 of *S*. *drobovii* var. *iskanderkulica* (Tzvel.) M. Nobis & A. Nowak, 21 of *S*. *ovczinnikovii* Roshev., 15 of putative hybrid *S*. *magnifica* × *S*. *caucasica*, and 25 of *S*. *lingua* × *S*. *caucasica*. Nomenclature of species follows Nobis *et al*. [40].

### Morphological measurements and multivariate analyses

For each well-developed specimen, 35 morphological characters (29 quantitative and six qualitative) were examined (Table 1). Each specimen was treated as an Operational Taxonomic Unit (OTU) in accordance with the methods used in numerical taxonomy [61]. Quantitative characters were visualized using boxplots generated in R [62]. For each character, the Shapiro-Wilk test was run in R using the MVN package [63] after all samples had been measured in order to determine the univariate normality distribution. The Pearson’s coefficient was implemented in PAST 4.03 [64] to display the correlation between characters. Levene’s test was used to determine whether variances are homogeneous, followed by a one-way ANOVA.

**Table 1:** Quantitative and qualitative characters for morphological examination.

Code	Morphological characters
Quantitative characters (in millimeters)	
AL	floret (= anthecium) length
AW	floret (= anthecium) width
DHC	dorsal hair length on callus
VHC	ventral hair length on callus
FL	callus foot ring length
FW	callus foot ring width
DHA	length of dorsal hairs on lemma
VHA	length of ventral hairs on lemma
CL	callus length
CH	corolla hair length
DDL	distance from the end of the dorsal line of hairs to the top of the lemma
DVL	distance from the end of the ventral line of hairs to the top of the lemma
Coll	column (lower segment of the awn) length
CW	column width
Seta	seta (upper segment of the awn) length
Awn	awn length
S/C	ratio of seta length to column length
HC	length of hair on column
HS	length of hair on seta
HS/HC	ratio of length: seta hair to column hair
Culm	culm length
LL	vegetative leaves length
Lig	length of ligule of vegetative leaves
UG	upper glume length
LG	lower glume length
LW	vegetative leaves width
LAH	length of hairs on adaxial surface of vegetative leaf
LLH	length of hairs on ligule of vegetative shoots
USW	upper culm’s sheath width
Qualitative characters	
NH	character of culm node (glabrous = 0, pilose = 1)
CS	character of culm sheaths (glabrous = 0, scabrous = 1, or pubescent = 2)
CAL	character of abaxial surface of vegetative leaves (glabrous = 0, scabrous = 1, or shortly pilose = 2)
FR	character of callus foot ring (pyriform = 1, goblet-like concave = 2, ovate = 3)
DS	character of dorsal surface of the callus (glabrous = 0, shortly pilose = 1, pilose = 2)
USC	character of upper culm sheath (glabrous = 0, scabrous = 1, or pubescent = 2)

The Principal Component Analyses (PCA) were performed in STATISTICA 13.3 [65] based on quantitative characters. Factor Analysis on Mixed Data (FAMD) was carried out in R using selected quantitative and qualitative characters. FAMD is a combination of PCA and multiple correspondence analysis (MCA; [66]). The existence of transgressive characters [67] was checked by calculating the mean value and standard error per quantitative character between *S*. *magnifica*, *S*. *lingua*, *S*. *caucasica* subsp. *nikolai*, and hybrids using PAST 4.03. The data were analyzed using one-way ANOVA followed by Tukey’s post hoc test as the assumptions of normality and homogeneity of variances were met or using the Kruskal-Wallis followed by Dunn’s test due to violations of the assumptions.

### Genomic library preparation, DArT sequencing and DArT data filtering

Molecular analyses were based on 37 samples (S1 Table), of which six represent *S*. *caucasica* Schmalh. subsp. *caucasica*, six of *S*. *caucasica* subsp. *nikolai* M. Nobis, A. Nobis & A. Nowak, four of *S*. *magnifica* A. Junge, two of *S*. *narynica* M. Nobis, two of *S*. *drobovii* var. *drobovii*, one of *S*. *drobovii* var. *iskanderkulica* (Tzvel.), three of *S*. *lingua* Roshev., three of *S*. *ovczinnikovii* Roshev., two of *S*. *glareosa* P.A. Smirn., five putative hybrids of *S*. *magnifica* × *S*. *caucasica*, and three putative hybrids of *S*. *lingua* × *S*. *caucasica*. *Stipa glareosa* P.A. Smirn. was selected as an outgroup following previous results on the phylogeny of *Stipa* described by Krawczyk *et al*. [68–70]. Whole genomic DNA was isolated using Genomic Mini AX Plant Kit (A&A Biotechnology, Poland). NanoDrop One (Thermo Scientific, USA) was used to perform the quantification check. Following the DArTseq methodology, each sample was diluted to a concentration of 50–100 ng/uL. The purified DNA (1–2 μg for each sample) was shipped to Diversity Arrays Technology Pty ltd (Canberra, Australia) for sequencing and marker identification.

DArTseq is a hybrid of DArT complexity reduction techniques with next-generation sequencing technologies. To pick the most appropriate complexity reduction strategy, the technology is tuned for each organism and application. Based on the results of testing several enzyme combinations for complexity reduction for *Stipa*, Diversity Arrays Technology Pty Ltd. chose the PstI-MseI approach. The reverse adapter contained a flowcell attachment region and MseI-compatible overhang sequence. Only “mixed fragments” (PstI-MseI) were effectively amplified by PCR using an initial denaturation step of 94 °C for 1 min, followed by 30 cycles with the following temperature profile: denaturation at 94 °C for 20 s, annealing at 58 °C for 30 s, and extension at 72 °C for 45 s, with an additional final extension at 72 °C for 7 min. After PCR, equimolar amounts of amplification products from each sample of the 96-well microtiter plate were bulked and applied to c-Bot (Illumina, USA) bridge PCR, followed by sequencing on Hiseq2500 (Illumina, USA). The sequencing (single read) was run for 77 cycles.

Sequences generated from each lane were analyzed utilizing proprietary DArT analytical pipelines. The fastq files were initially processed in the primary pipeline to remove low-quality sequences, with the barcode region receiving more strict selection criteria than the rest of the sequence. As a result, the "barcode split" assignment of sequences to individual samples was quite reliable. Approximately 2.5 million sequences were found and utilized in marker calling for each barcode/sample.

### SNPs data analysis

For the downstream analyses, we applied co-dominant single nucleotide polymorphisms (SNPs) markers, which were analyzed using RStudio package “dartR” [71] and “devtools” [72]. Data filtering includes the following steps: 1) removing all monomorphic loci, 2) calculating call rates for each locus (threshold 95%), 3) removing loci with reproducibility below predetermined threshold (<1), 4) removing secondaries form, keeping only one random sequence tag per SNP locus, and 5) filtrating MAF loci (threshold 5%). Maximum Likelihood (ML) tree was generated based on 4,653 SNP loci (processed using R) for 37 samples of ten taxa (*S*. *caucasica* subsp. *caucasica*, *S*. *caucasica* subsp. *nikolai*, *S*. *lingua*, *S*. *magnifica*, *S*. *drobovii*, *S*. *narynica*, *S*. *ovczinnikovii*, putative hybrid of *S*. *magnifica* × *S*. *caucasica* and *S*. *lingua* × *S*. *caucasica* and *S*. *glareosa* as outgroup). The genlight object was converted to the FASTA file (package dartR), and heterozygous locations were replaced with standard ambiguity codes. The FASTA file was then analyzed using MEGA version 11.0.13 [73], with the GTR (General Time Reversible) model chosen as the best fitting substitution model based on AIC values, and the bootstrap method as the phylogenetic test with 500 replications. To answer the question of which subspecies of *S*. *caucasica* was involved in the hybridization process, we provided two separate STRUCTURE analyses to visualize the genetic proportion of *S*. *magnifica*, *S*. *caucasica* subsp. *caucasica*, and *S*. *caucasica* subsp. *nikolai* as well as *S*. *lingua*, *S*. *caucasica* subsp. *caucasica*, and *S*. *caucasica* subsp. *nikolai* and their putative hybrids respectively, using SNPs markers. In total 21 samples (6,413 SNP markers) were utilized for STRUCTURE analysis in hybrid detection of *S*. *magnifica* × *S*. *caucasica* and 18 samples (5,053 SNPs) in *S*. *lingua* × *S*. *caucasica* analysis. For the analyses we set burnin period = 10,000 for the first and 50,000 for second set of taxa and MCMC reps after burnin = 20,000 and 50,000 for respectively first and second set with 20 independent runs each for K = 1–4.

DIYABC Random Forest v1.0, which has two applications—scenario simulations utilizing SNPs data and Random Forests using SML algorithms—was used to simulate custom evolutionary scenarios. It is accompanied by a number of statistical tests that assess the robustness and accuracy of conclusions [74]. In total, six samples of *S*. *caucasica* subsp. *nikolai*, six of *S*. *caucasica* subsp. *caucasica*, three of *S*. *lingua*, five of *S*. *magnifica*, three of *S*. *lingua* × *S*. *caucasica* s. lato, and four of *S*. *magnifica* × *S*. *caucasica* s. lato were analyzed (21 samples for checking *S*. *magnifica* × *S*. *caucasica* s. lato scenarios and 18 samples for *S*. *lingua* × *S*. *caucasica* s. lato scenarios). The number of loci we utilized was the same as in the STRUCTURE analysis respectively for each set of the taxa. For every scenario involving the emergence of *S*. *magnifica* × *S*. *caucasica* s. lato and *S*. *lingua* × *S*. *caucasica* s. lato, a total of 10,000 simulations per scenario were conducted. The historical parameter priors for all population number (Ne) estimations were set at 10–100,000, and the time (t) parameter was set at 10–500,000. Time estimation (years) was set as the range between 2–4 years (in the case of perennial plants such as *Stipa* this time may vary between species and individuals).

The evolutionary connections and historical mixing events across populations were inferred using TreeMix [75] based on 3,187 SNPs data from 37 individuals belonging to ten taxa (the same individuals as in Maximum Likelihood, generated after adding one step of filtration of calculating call rates for each locus (threshold 100%) after MAF loci filtration to left only loci with non-missing data). Similarly to the ML, *S*. *glareosa* was used for rooting the tree. Data were processed assuming the independence of all SNPs with a window size of one SNP (k = 1), and a range of migration events (m) from 1 to 10 were tested. We estimated the optimal number of migration edges by using an ad hoc statistic based on the second-order rate of change in log likelihood implemented in OptM v. 0.1.6 [76].

#### Nomenclature

The electronic version of this article in Portable Document Format (PDF) in a work with an ISSN or ISBN will represent a published work according to the International Code of Nomenclature for algae, fungi, and plants, and hence the new names contained in the electronic publication of a PLOS ONE article are effectively published under that Code from the electronic edition alone, so there is no longer any need to provide printed copies.

In addition, new names contained in this work have been submitted to IPNI, from where they will be made available to the Global Names Index. The IPNI LSIDs can be resolved and the associated information viewed through any standard web browser by appending the LSID contained in this publication to the prefix http://ipni.org/. The online version of this work is archived and available from the following digital repositories: PubMed Central, LOCKSS.

## Results

### Morphology-based species delimitation and hybrids identification

The Principal Component Analysis (PCA) of 11 taxa of *Stipa* was computed using 11 quantitative characters. The first three factors explained 89.4% of the total morphological variance (55.66%, 25.45%, and 7.91%). In total, seven characters were associated with the first axis and four with the second axis, where nine of the characters showed positive factor loading (>0.6) and two characters showed negative factor loading (<-0.6) on the three first axes (S2 Table). Factor Analysis on Mixed Data (FAMD) carried out by combining 11 quantitative characters and three qualitative characters, explained 58.39% of variation for the first three components (32.66%, 16.7%, and 9.03%), respectively. The results of the one-way analysis of variance (ANOVA) for individual characters (refer to S2 Table) revealed significant differences with a p-value <0.05.

**Fig 1:**
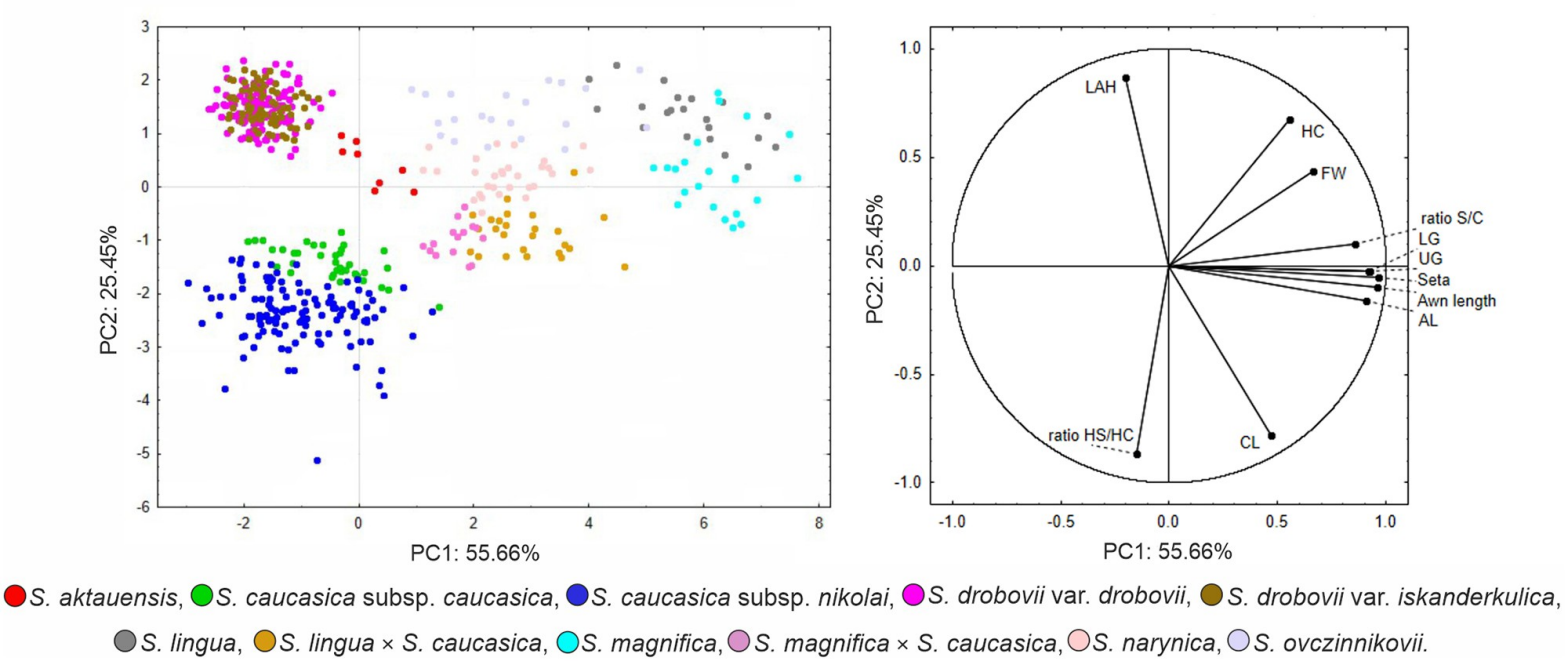
The PCA analysis performed on 14 quantitative characters of 11 *Stipa* taxa having unigeniculate and throughout pilose awns.

The PCA analysis reveals a few groups of OTUs with slightly or completely overlapping clouds of OTUs corresponding to *S*. *caucasica* subsp. *caucasica* and *S*. *caucasica* subsp. *nikolai* as well as *S*. *drobovii* var. *drobovii* and *S*. *drobovii* var. *iskanderkulica* (Fig 1). The OTUs of both putative hybrids (*S*. *magnifica × S*. *caucasica* and *S*. *lingua* × *S*. *caucasica*) were clearly delimited from their putative parental taxa.

**Fig 2:**
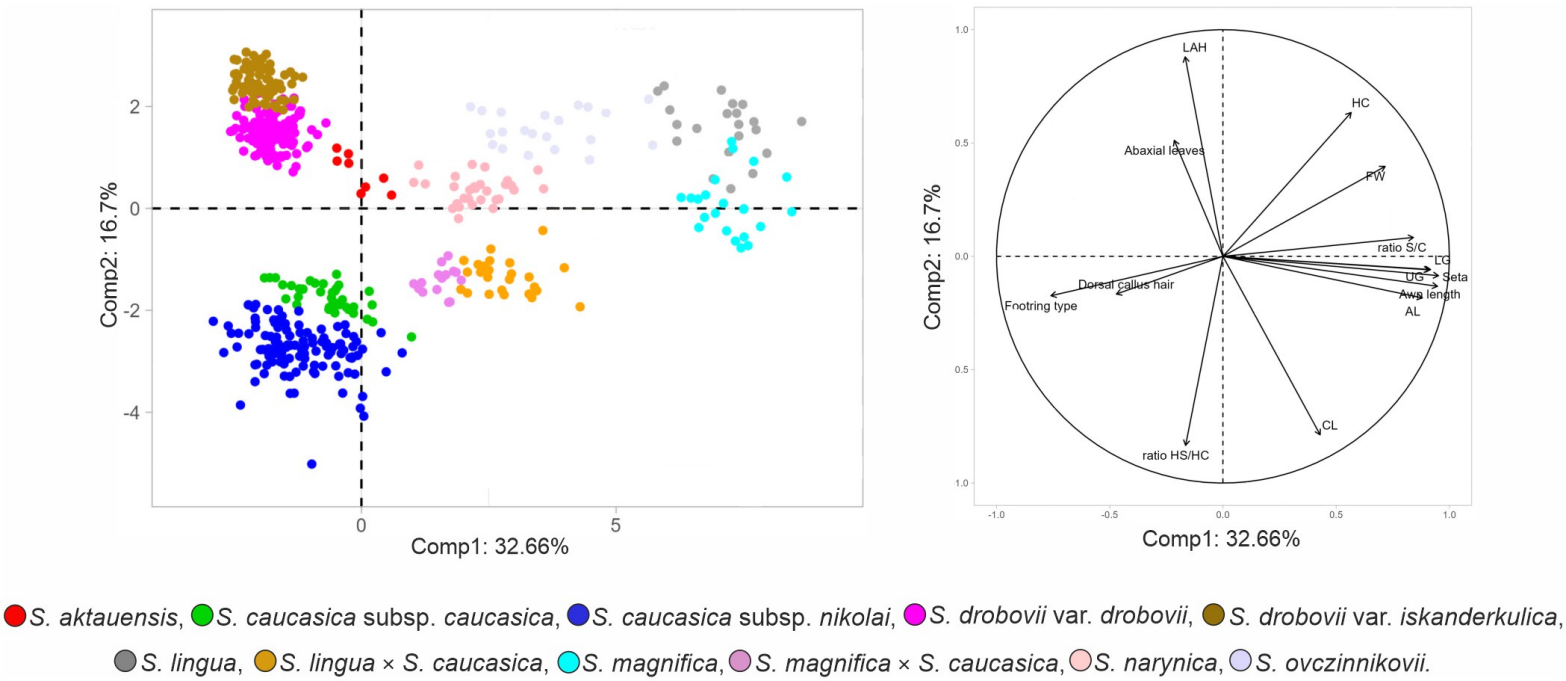
The FAMD analysis performed on 11 quantitative characters and 3 qualitative characters of 11 *Stipa* taxa.

FAMD analysis based on quantitative and qualitative morphological features allows for a better delimitation of OTUs of the studied taxa (Fig 2). Although there are still some OTUs of *S*. *drobovii* var. *iskanderkulica* and *S*. *drobovii* var. *drobovii* that overlap, FAMD was more successful in separating these two taxa than the prior PCA (Fig 1) where the two taxa overlapped with each other. This was made possible by the addition of the character of the abaxial surface of vegetative leaves, which resolved the confusion between these two taxa clumped together (*S*. *drobovii* var. *drobovii* has glabrous vs. *S*. *drobovii* var. *iskanderculica* has pubescent abaxial surface of leaves).

**Fig 3:**
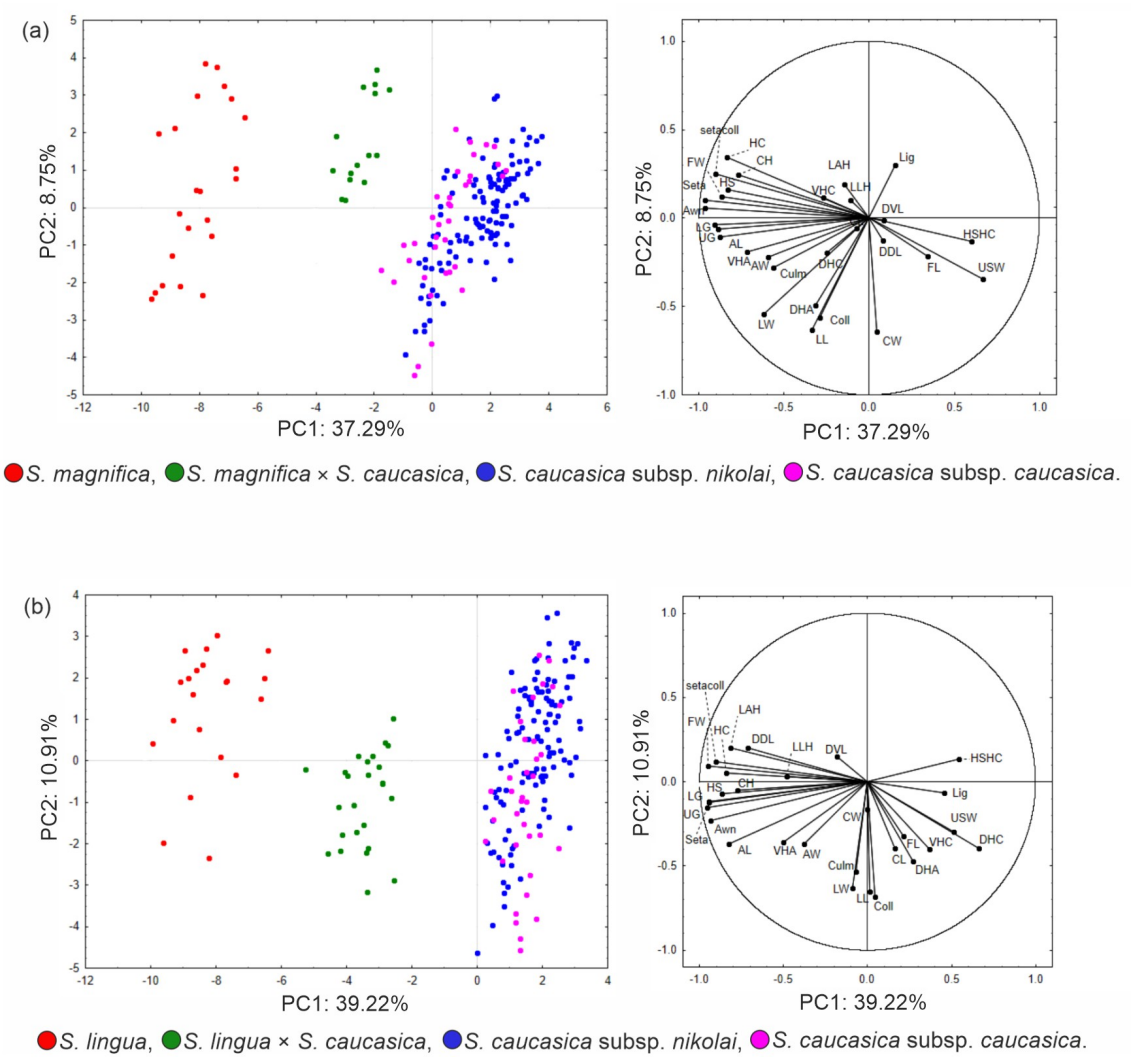
The PCA analyses performed on 29 quantitative characters of selected specimens of feathergrasses. (a) *S*. *magnifica*, *S*. *caucasica* subsp. *caucasica*, *S*. *caucasica* subsp. *nikolai*, and *S*. *magnifica* × *S*. *caucasica* s. lato; (b) *S*. *lingua*, *S*. *caucasica* subsp. *caucasica*, *S*. *caucasica* subsp. *nikolai*, and *S*. *lingua* × *S*. *caucasica* s. lato.

Since the PCA revealed several slightly overlapping group of OTUs, we decided to present two separate PCA analyses showing clear intermediacy of *S*. *magnifica × S*. *caucasica* s. lato and *S*. *lingua × S*. *caucasica* s. lato, respectively with their parental taxa. The first analysis (Fig 3a), based on 29 quantitative morphological characters, revealed separated clouds of OTUs of *S*. *magnifica*, *S*. *caucasica*, and their putative hybrid. The first three factors explained 53.96% of the total variation (37.29%, 8.75%, and 7.92%; S3 Table). The analysis showed 13 characters studied correlated with first axis, two with the second axis, and one with the third axis. Two characters had high positive factor loadings (>0.6) on the three first axes and 14 had high negative factor loadings (<-0.6; S3 Table). This putative hybrid is similar to *S*. *caucasica* by having a relatively narrow foot ring of the callus as well as similar in some characters to *S*. *magnifica* by having long hairs on the column and a straight seta.

The PCA performed on 29 quantitative morphological characters of *S*. *lingua*, *S*. *caucasica* subsp. *caucasica*, *S*. *caucasica* subsp. *nikolai*, and their putative hybrids explained 57.53% variation for the first three factors (39.22%, 10.91%, 7.40%; S4 Table). The analysis showed 13 characters associated with the first axis and three with the second axis. In total, one character had high positive factor loadings (>0.6) on the three first axes and 15 had high negative factor loadings (<-0.6; S4 Table). The analysis reveals an intermediate position of OTUs representing putative hybrids between *S*. *caucasica* s. lato and *S*. *lingua* (Fig 3b). Boxplots diagrams for selected quantitative characters of putative hybrids and parental taxa can be seen in S1 Fig.

### SNP-based species delimitation and hybrids identification

**Fig 4:**
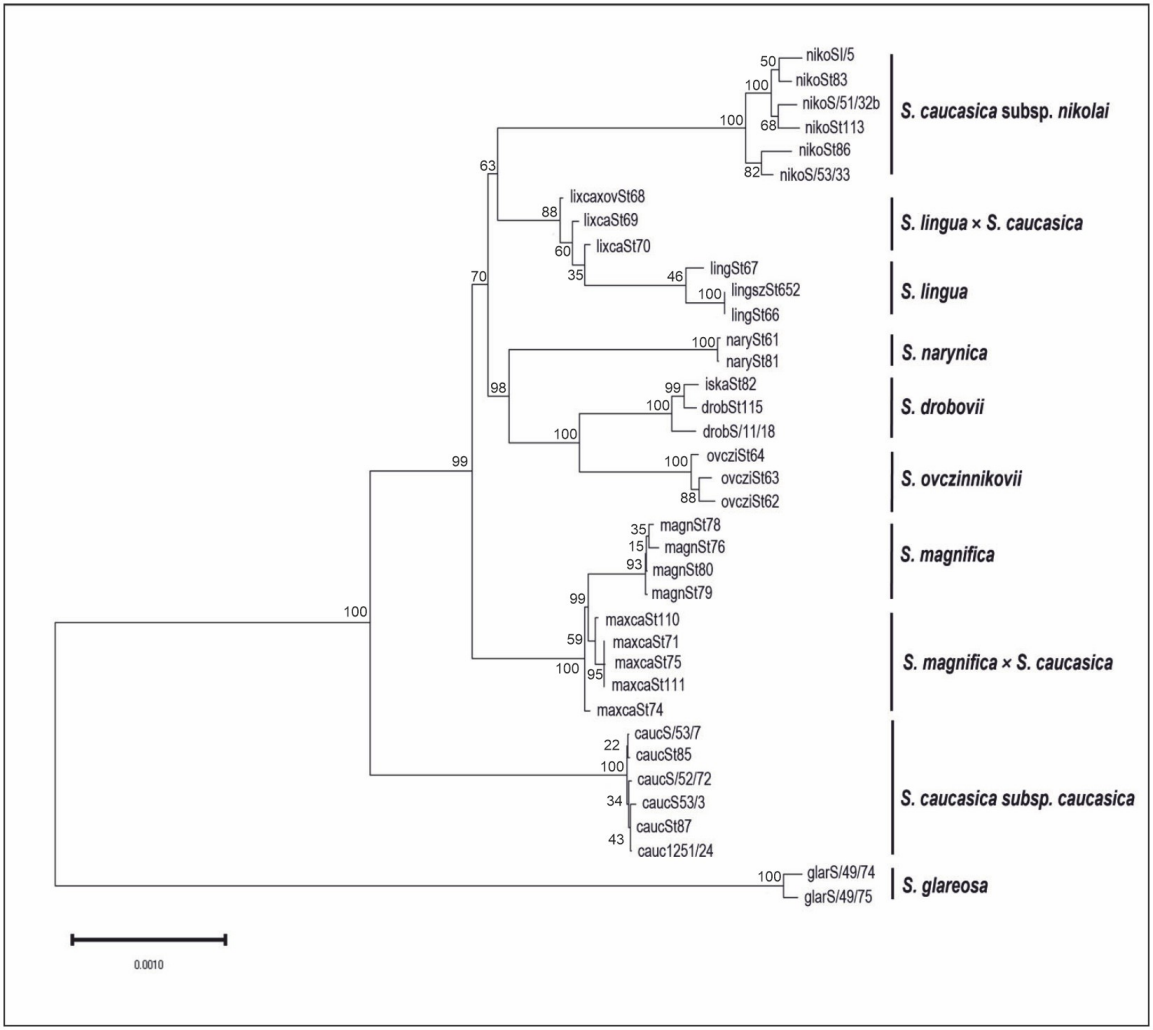
Maximum Likelihood tree based on SNPs markers from DArTseq analysis of 10 taxa of *Stipa* sect. *Smirnovia*. List of samples are given in S1 Table.

The Maximum Likelihood (Fig 4) revealed five clusters: cluster I consists of *S*. *caucasica* subsp. *nikolai*, the putative hybrid of *S*. *lingua* × *S*. *caucasica* s. lato and *S*. *lingua*; cluster II consists of *S*. *narynica*, *S*. *drobovii* (*S*. *drobovii* var. *drobovii* and *S*. *drobovii* var. *iskanderkulica*) and *S*. *ovczinnikovii*; cluster III consist of *S*. *magnifica* and *S*. *magnifica* × *S*. *caucasica* s. lato; cluster IV consist of *S*. *caucasica* subsp. *caucasica*; and cluster V of *S*. *glareosa*.

**Fig 5:**
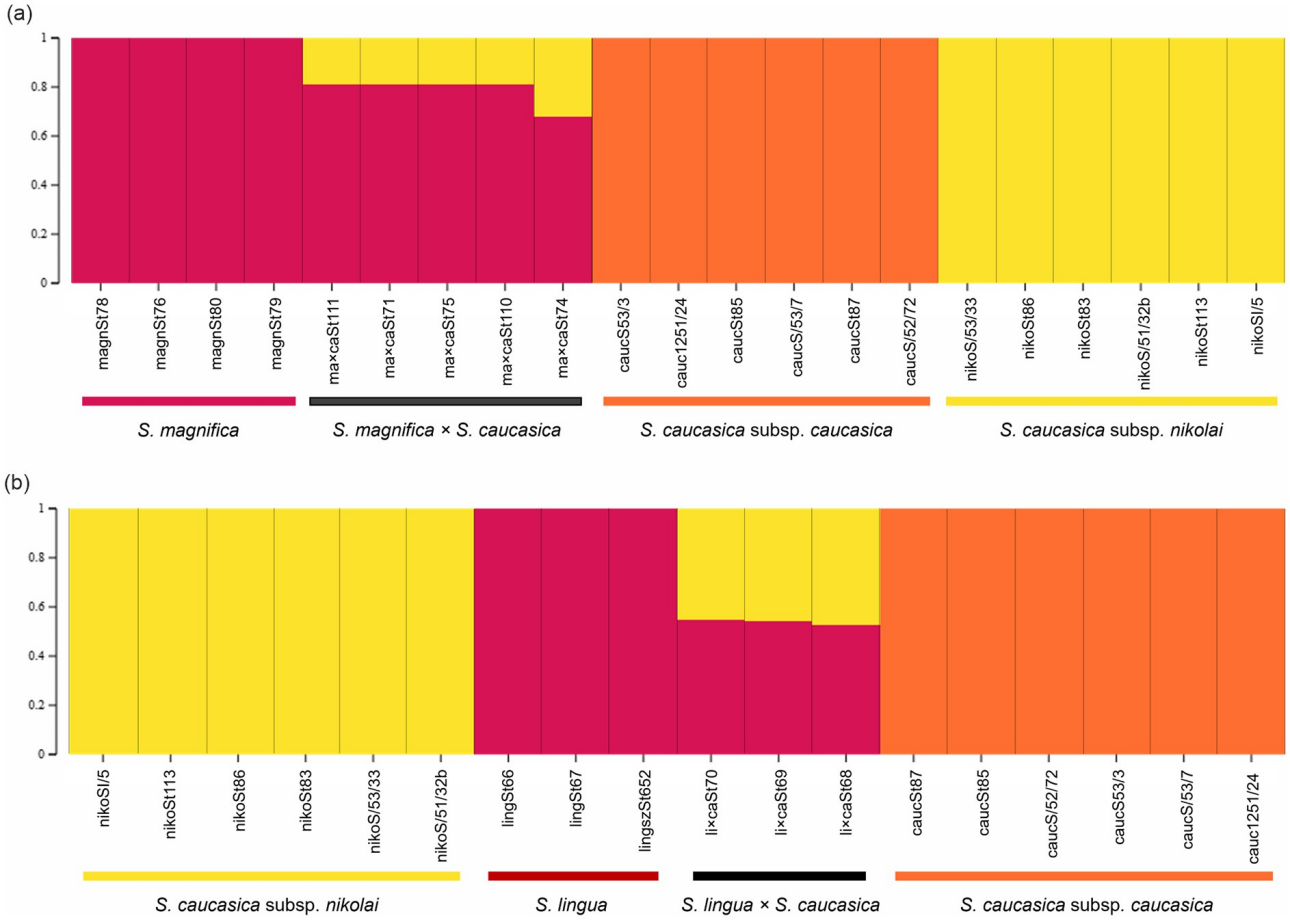
The results of STRUCTURE analysis based on SNPs markers. (a) *S*. *caucasica* subsp. *caucasica*, *S*. *caucasica* subsp. *nikolai*, *S*. *magnifica*, and *S*. *magnifica × S*. *caucasica* (K = 3), and (b) *Stipa caucasica* subsp. *caucasica*, *S*. *caucasica* subsp. *nikolai*, *S*. *lingua*, and *S*. *lingua × S*. *caucasica* (K = 3). List of samples are given in S1 Table.

The STRUCTURE analysis of *S*. *caucasica* subsp. *caucasica*, *S*. *caucasica* subsp. *nikolai*, *S*. *magnifica*, and putative hybrid *S*. *magnifica × S*. *caucasica* s. lato based on 21 samples revealed K = 3 as the best K value (Fig 5a). Individuals marked as potential hybrids of *S*. *magnifica × S*. *caucasica* s. lato (ma*×*caSt110, ma*×*caSt74, ma*×*caSt75, ma*×*caSt71, ma*×*caSt111) showed an unequal admixture of two species, *S*. *magnifica* and *S*. *caucasica* subsp. *nikolai*. One individual (ma*×*caSt74) showed admixture of 68% of *S*. *magnifica* and 32% of *S*. *caucasica* subsp. *nikolai*, whereas the remaining four putative hybrid samples had around 80% of *S*. *magnifica* genetic admixture and less than 20% of *S*. *caucasica* subsp. *nikolai*. Given the evidence of a shared genetic fraction of the two parental taxa that is less than 50:50 in *S*. *magnifica × S*. *caucasica* subsp. *nikolai* hybrid, it raises the probability that the samples are the result of a backcrossings with *S*. *magnifica*.

Whereas the STRUCTURE analysis based on 18 samples of *S*. *lingua*, *S*. *caucasica* subsp. *caucasica*, *S*. *caucasica* subsp. *nikolai*, and putative hybrid *S*. *lingua × S*. *caucasica* s. lato exhibited the best K value = 3 (Fig 5b). The three putative hybrid samples of *S*. *lingua × S*. *caucasica* (li*×*caSt68, li*×*caSt70, and li*×*caSt69) showed an admixture of approximately 50:50 proportions for both *S*. *caucasica* subsp. *nikolai* and *S*. *lingua*, implying that they are F1 hybrids of these two above mentioned taxa.

To check the existence of transgressive characters in *S*. *magnifica × S*. *caucasica*, three quantitative characters (awn length, seta (upper segment of the awn) length, and length of hair on seta) were analyzed using one-way ANOVA followed by Tukey’s post hoc test, while the rest 26 quantitative characters were analyzed using non-parametric Kruskal-Wallis and followed by Dunn’s post hoc test. This calculation defines two characters of the hybrid *S*. *magnifica* × *S*. *caucasica* subsp. *nikolai* (ventral hair length on callus and length of hairs on ligule of vegetative shoots) as positive transgressive and two characters as negative (distance from the end of the ventral line of hairs to the top of the lemma and distance from the end of the dorsal line of hairs to the top of the lemma), whereas six characters were classified as intermediate between parental taxa. In nine characters, the hybrid is classified as *magnifica*-like and five as *nikolai*-like. Additionally, two characters of the hybrid were classified as parental codominance, where the mean value of the hybrid is not significantly different from the putative parental taxa, but the mean values of the two parents are significantly different from each other, and three characters as not significant (S5 Table).

One quantitative character, the length of the column (lower segment of the awn), was examined using one-way ANOVA and Tukey’s post hoc test to determine whether transgressive characters exist in *S*. *lingua* × *S*. *caucasica*. The remaining 28 quantitative characters were examined using non-parametric Kruskal-Wallis and Dunn’s post hoc test. The hybrid *S*. *lingua* × *S*. *caucasica* subsp. *nikolai* shows positive transgressive in one character (column width). The hybrid has one character classified as intermediate in comparison with both parental taxa, whereas 13 characters were classified as *lingua*-like, four as *nikolai*-like, two characters as codominance, and eight as not significant (S6 Table).

### Evolutionary scenarios and historical mixing events

**Fig 6:**
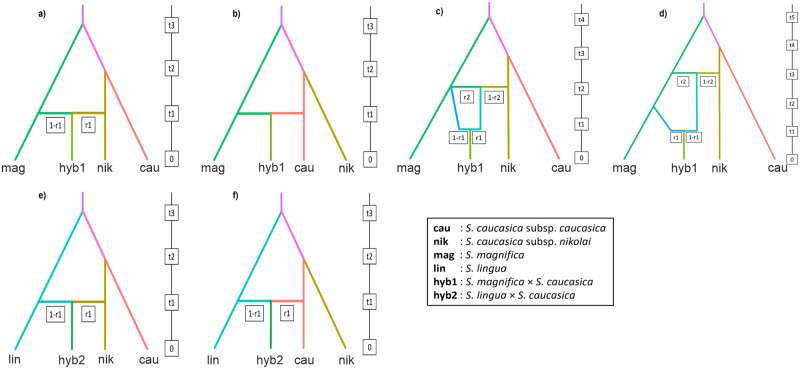
DIYABC analysis of four scenarios for the emergence of two hybrids of *S*. *magnifica × S*. *caucasica* and *S*. *lingua* × *S*. *caucasica*.

STRUCTURE analysis detected one of the hybrid samples of *S*. *magnifica* × *S*. *caucasica* as an F1 hybrid and four as backcross hybrids, while all hybrid samples of *S*. *lingua* × *S*. *caucasica* are F1 hybrids. We tested six possible evolutionary scenarios for the emergence of hybrids using the DIYABC analysis (four scenarios for *S*. *magnifica* × *S*. *caucasica* and two scenarios for *S*. *lingua* × *S*. *caucasica*). Evolutionary scenarios for *S*. *magnifica* × *S*. *caucasica* are described as following: i) the putative hybrids are F1 hybrids of *S*. *magnifica* and *S*. *caucasica* subsp. *nikolai* (Fig 6a/scenario 1) or *S*. *magnifica* and *S*. *caucasica* subsp. *caucasica* (Fig 6b/scenario 2), or ii) whether putative hybrids are backcross hybrids (scenario 3 and 4). The results of the first two scenarios were not statistically significant, since the observed data did not fit either of the two selected scenarios according to the Linear Discriminant Analysis (S2 Fig). Scenario 3 shows that the population of *S*. *magnifica* (N4) hybridized with *S*. *caucasica* subsp. *nikolai* at t3, and the hybrids backcrossed with the *S*. *magnifica* populations (N6) that emerged at the same time as the hybridization took place (Fig 6c). In scenario 4, the backcross event with *S*. *magnifica* (N6) appeared afterward (t2) the gene flow between *S*. *magnifica* (N4) and *S*. *caucasica* subsp. *nikolai* (Fig 6d). The obtained result supports that the observed data fall into scenario 4 as the Linear Discriminant Analysis showed posterior probability of 0.585 (S3 Fig).

Additionally, the occurrence of hybrids *S*. *lingua* × *S*. *caucasica* based on DIYABC carried out by two scenarios involving hybridization of i) *S*. *lingua* and *S*. *caucasica* subsp. *nikolai* (Fig 6e/scenario 5) or ii) *S*. *lingua* and *S*. *caucasica* subsp. *caucasica* (Fig 6f/scenario 6). Analysis results reveals that the hybrids originated from hybridization between *S*. *caucasica* subsp. *nikolai* and *S*. *lingua* (Fig 6e) instead of *S*. *caucasica* subsp. *caucasica* and *S*. *lingua* (Fig 6f). The results of posterior probability and linear discriminant analysis show that the observed data falls into the first scenario (posterior probability: 1; S4 Fig).

**Fig 7:**
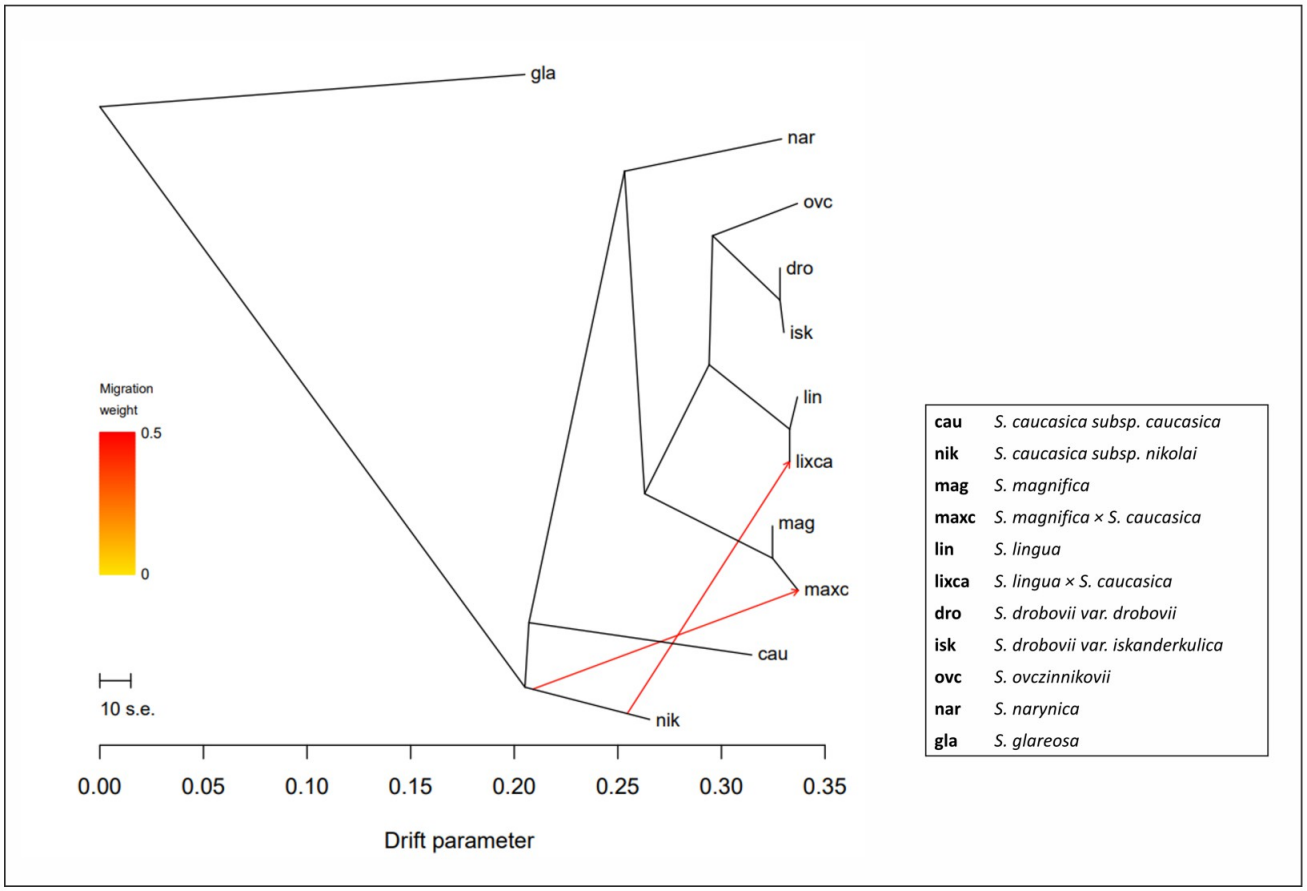
Maximum likelihood tree inferred with Treemix showing the most likely migration event (m = 2). The direction of gene flow is represented with an arrow colored according to the percentage of alleles (weight) originating from the source.

In the DIYABC analysis, median parameter of time for the chosen scenario revealed that, the hybridization between *S*. *magnifica* and *S*. *caucasica* subsp. *nikolai* (t3) was about 161864 generations ago (which means ca. 0.647–0.323 mya, assuming that the range of generation time is 2 to 4 years; S7 Table) with the admixture rate (r2) of around 0.32 for *S*. *magnifica* and 0.68 for *S*. *caucasica* subsp. *nikolai*. The backcross event (t1) occurred about 5,673.46 generations ago with a population admixture rate (r1) of around 0.61 for *S*. *magnifica* and 0.39 for *S*. *caucasica* subsp. *nikolai*. According to this model, population diversification of *S*. *magnifica* (t2) occurred about 23,724.1 generations ago. The hybridization process between *S*. *lingua* and *S*. *caucasica* subsp. *nikolai* (t1) based on chosen scenario (Fig 6e) occurred about 6,132.97 generations ago (ca. 0.024–0.012 mya, assuming the range of generation time is 2–4 years; S8 Table) with a rate of population admixture of about 0.45 for *S*. *caucasica* subsp. *nikolai* and 0.55 for *S*. *lingua*. The TreeMix supported the results of previous analyses and indicated two migration events (characterized by similar, strong migration weight) from *S*. *caucasica* subsp. *nikolai* to both putative hybrids (Fig 7).

### Taxonomic implications

Below, we present detailed descriptions of the two new natural hybrids: *Stipa × muksuensis* (= *S*. *lingua × S*. *caucasica* subsp. *nikolai*) and *Stipa × ochyrae* (= *S*. *magnifica × S*. *caucasica* subsp. *nikolai*). For schematic figures with explanations of the morphological traits characterized below, please see e.g. Nobis et al. [40].

#### Stipa × muksuensis

M. Nobis, Klichowska, A. Nowak, P. Sinaga, *nothosp*. *nov*. (*S*. *lingua* A. Junge *× S*. *caucasica* subsp. *nikolai* M. Nobis, A. Nobis & A. Nowak) [urn:lsid:ipni.org:names:XXXXX] (S5 Fig). Type: Tajikistan, Sartala settl., near Karakendzhe (Kysyl-Suu River valley), steppe grassland / tall herb meadow on the left slope of Muksu River valley, 39°14ʹ58.01ʹʹN / 71°26ʹ14.03ʹʹ E, elev. 1930 m, slope 45 incl. NW, 3 June 2015, wp. 514, *M*. *Nobis*, *A*. *Nowak* (holotype KRA 0453433, isotypes KRA 4533432, KRA 453408, KRA 453435, KRA 453436, KRA 435434).

*Description*. Plants perennial, densely tufted, with a few culms and numerous vegetative shoots; culms 30–56 cm tall, 2-3-noded, pubescent at nodes and shortly pubescent (rarely glabrous) below them. Leaves of vegetative shoots: sheaths shortly pubescent or scabrous, at margins white and ciliate, cilia 0.5–1.5 mm long; ligules truncate, 0–0.2 mm long and with 0.6–2.2 mm long and dense hairs at the apex; blades convolute, 10–35 cm long, 0.8–1.1(–1.3) mm in diam., upper (adaxial) surface densely short-pilose with hairs 0.10–0.20(–0.25) mm long, glabrous and smooth beneath (abaxially). Cauline leaves: sheaths scabrous to very shortly pubescent, rarely glabrous, margins white and ciliate (1.0–1.5 mm long), sheaths usually shorter than internodes, upper sheath up to 25 cm long, scabrous or glabrous, uninflated or inflated; ligules 0.1–0.3 mm long, truncate, at apex with 0.3–1.5 mm long hairs; blades glabrous and smooth, up to 15 cm long, the upper one on the culm 3–7 cm long. Panicle 8–15 cm, contracted, with 5 to 9 spikelets, at the base enclosed by sheath of uppermost leaf, branches erect, setulose, single or paired. Glumes subequal, lower glume (65–)70–85(–90) mm long, upper glume (64–)65–85(–88) mm, lanceolate, tapering into long hyaline apex, glabrous or along the middle vein setulose with cilia up to 1 mm long. Floret (callus + lemma) (13–)14.0–15.0(–15.5) long and 0.8–1.2 mm wide. Callus (1.8–)2.0–2.2 mm long, densely and long pilose, ventrally with the longest hairs 1–1.4 mm long, dorsally with straight hairs 0.7–1.0 mm long, occurring only in the middle part of the callus, base of callus slightly expanded, peripheral ring 0.35–0.45(–0.50) mm in diam., broadly cuneate, scar elliptic. Lemma pale green, on dorsal surface with abundant hooks and 7 lines of ascending hairs, 1.0–1.5 mm long, ventral lines terminating 1–2 mm below the top of lemma, dorsal line terminating 1–4 mm below the top of lemma; lemma below the apex scabrous due to prickles and short hairs, surpassed by a well-developed ring of unequal hairs (0.5–)1.0–1.5(–2.0) mm long at the top. Palea equal to lemma in length, glabrous or with a dorsal line of scattered hairs, up to 1 mm long. Awn 160–200 mm long, unigeniculate; column 23–31 mm, twisted, 0.5–0.7 mm wide near the base, shortly and densely pilose, with hairs (0.7–)1.0–1.5(–2.0) mm long, in the upper part gradually increasing in length toward geniculation; seta 130–172 mm long, straight, 4.8–5.5 times longer than column, hairs in lower part of seta 7.5–9.2 mm long, gradually decreasing in length toward the apex. Anthers yellow, 6–7 mm, glabrous.

*Paratype*. Tajikistan, East Tajikistanian A subregion, Kyzyl-su River Valley, ca. 168 km WNW of Karakul Lake, near Kaszat, steppe, 39°17’15.46"N / 71°24’32.65"E, elev. 1979 m, 14 Jul 2021, wp. 1469, *M*. *Nobis*, *E*. *Klichowska* (KRA 0594243, KRA 0594241, KRA 0594244, KRA 0594246, KRA 0594355, KRA 0594354); East Tajikistanian A subregion, Kyzyl-su River Valley, S of Alga settl., ca. 14 km NE of Damburacha, steppe on the hill, 39°19’39"N / 71°32’49"E, elev. 2102 m, 16 Jul 2021, *M*. *Nobis*, *E*. *Klichowska* (KRA 0594865).

*Phenology*. Flowers from May to June.

*Habitat*. Steppe grasslands and high mountain steppes at elevation between 1800–2100 m.

*Distribution*. Northern Tajikistan (Peter the First Mts, Hissar Mts), within Muksu and Kara-Suu River valleys.

*Etymology*. This species is named for the Muksu river valley in northern Tajikistan.

#### Stipa × ochyrae

M. Nobis & Klichowska, *nothosp*. *nov*. (*S*. *magnifica* A. Junge *× S*. *caucasica* subsp. *nikolai* M. Nobis, A. Nobis & A. Nowak) [urn:lsid:ipni.org:names:XXXXX] (S6 Fig). Type: Kyrgyzstan, Western Tian-Shan, Alai, steppe grasslands on chalk hill, near the road in the E part of Tash-Koroo settl., ca. 9 km SW of Gulcha, 40°15ʹ0.8ʹʹN / 73°23ʹ0.8ʹʹ E, elev. 1924 m, slope 35 incl. S-SE, 2 Jul 2015, wp. 575, *M*. *Nobis*, *A*. *Nowak* (holotype KRA 0486046, isotypes KRA 0486045, KRA 0486047, KRA 0486048, KRA 0486049, KRA 0486050, KRA 0486051).

*Description*. Plants perennial, densely tufted, with a few culms and numerous vegetative shoots; culms 35–55 cm tall, 2-3-noded, glabrous or rarely sparsely pubescent at nodes and below them. Leaves of vegetative shoots: sheaths glabrous and smooth, at margins white and ciliate, cilia 0.4–1.5 mm long; ligules truncate, up to 0.1 mm long and densely hairy at the apex or completely reduced and replaced by a line of very dense and unequal 1–3 mm long hairs; blades convolute, 8–20 cm long, (0.6–)0.7–0.9(–1.1) mm in diam., upper (adaxial) surface densely shortly-pilose with hairs 0.10–0.15 mm long, glabrous and smooth beneath (abaxially). Cauline leaves: sheaths glabrous and smooth, margins white and ciliate (0.5–1.2 mm long), sheaths usually shorter than internodes, upper sheath up to 15 cm long, glabrous, uninflated; ligules 0.5–1.3 mm long, rounded, at apex and on the back with 0.5–1.5 mm long hairs; blades glabrous and smooth, up to 10 cm long, the upper one on the culm 3–5 cm long. Panicle 8–15 cm long, contracted, with 6 to 10 spikelets, at the base enclosed by sheath of uppermost leaf, branches erect, setulose, single or paired. Glumes subequal, lower glume 45–55 mm long, upper glume 44–54 mm, lanceolate, tapering into long hyaline apex, glabrous or rarely with scattered cilia along the middle vein. Floret (callus + lemma) 11.0–12.5 long and 0.8–1.1 mm wide. Callus (1.9–)2.0–2.3 mm long, densely and long pilose, ventrally with hairs 1.0–1.3 mm long, dorsally with straight hairs 0.8–1.0 mm long, base of callus cuneate, peripheral ring 0.2–0.3 mm in diam., scar elliptic. Lemma pale green, on dorsal surface with abundant hooks and 7 lines of ascending hairs, 1.0–1.5 mm long, ventral lines reaching the top or terminating 0.2–1.5 mm below top of lemma, dorsal line terminating 0.5–1.5 mm below top of lemma; lemma below the apex scabrous due to prickles and short hairs, surpassed by a well-developed ring of unequal hairs 1–2 mm long at the top. Palea equal to lemma in length, glabrous. Awn (125–)140–160(–170) mm long, unigeniculate; column 24–29 mm, twisted, 0.5–0.6 mm wide near the base, densely pilose, with hairs (1.0–)1.5–2.6 mm long, in the upper part gradually increasing in length toward geniculation; seta (100–)111–132(–138) mm long, straight, (4.4–)4.6–5.5(–6.0) times longer than column, hairs in lower part of seta 6.5–7.5 mm long, gradually decreasing in length toward apex. Anthers yellow, 5.5–6.5 mm, glabrous.

*Paratypes*. Kyrgyzstan, ca. 54 km to SE from Osh city and ca 8.5 km to the SW from Gulcho, near the road M41, steppe., 40°15ʹ0.8ʹʹN / 73°23ʹ0.8ʹʹ E, elev. 1978 m, 23 Jul 2016, wp. 718, *M*. *Nobis*, *A*. *Nobis* (KRA 00626825, KRA 00626826); Kyrgyzstan, NE Alai Range, ca. 57 km to the SE from Osh city, between Gulcho and Tashkoro vill., near the road M41, steppe., 40°15ʹ5.72ʹʹN / 73°23ʹ5.15ʹʹ E, elev. 1949 m, slope 30–40 incl. SE, 2 Jul 2017, wp. 897, *M*. *Nobis*, *E*. *Klichowska*, *A*. *Wróbel*, *A*. *Nowak* (KRA 474686, KRA 474690).

*Phenology*. Flowers from May to June.

*Habitat*. Steppe grasslands and high mountain steppes at elevation between 1900–2000 m.

*Distribution*. Southern Kyrgyzstan, within Dzhaily-Suu and Gulcha River valleys.

*Etymology*. This species is named in honour of prof. dr hab. Ryszard Ochyra, the eminent Polish botanist, bryologists, taxonomist and phytogeographer, of the W. Szafer Institute of Botany, Polish Academy of Sciences in Krakow.

## Discussion

Natural hybridization is recognized as an important evolutionary force in plants [1, 77] and may lead to several very important consequences, such as increase of the intraspecific genetic diversity, creation of new species, species extinction through genetic assimilation, or creation of new invasive genotypes [52]. Proper identification of hybrid individuals is crucial for evolutionary biology, ecology, conservation, and biogeography, as well as for understanding biodiversity. While traditional morphology-based taxonomy is valuable for studying morphological diversity, an integrated taxonomy approach is considered essential to address the complexities of species biology [78]. By combining morphological measurements and molecular data, an integrated taxonomy approach has been used to confirm and document many hybrid individuals and hybrid taxa [6, 10, 53].

In this research, we found evidence of natural hybridization among closely related taxa occurring in the high mountain steppes of Central Asia. In total, two positive transgressive characters and two negative transgressive characters were detected in *S*. *magnifica × S*. *caucasica* subsp. *nikolai*, here and below named as *S*. *× ochyrae*, while one positive transgressive morphological characters were detected in *S*. *lingua × S*. *caucasica* subsp. *nikolai*, here and below named as *S*. *× muksuensis*. The occurrence of more transgressive characters in *S*. *magnifica × S*. *caucasica* (which most likely arose via backcrossing) compared to *S*. *lingua × S*. *caucasica* (an F1 hybrid) is in line with the findings of Rieseberg and Ellstrand [79], who confirmed a higher proportion of extreme morphological characters in later generation hybrids compared to the first generation (10.2% and 30.6%, respectively). This phenomenon is explained by an increase in hybrid mutation rates, heterosis, a concentration of recessive alleles in heterozygous, and a decrease in developmental stability [79]. The establishment of transgressive traits allows hybrids to occupy extremely severe areas that are different from those occupied by their parental taxa [67]. We found a greater dominance of morphological characters in *S*. *lingua* × *S*. *caucasica* from *S*. *lingua* versus to *S*. *caucasica* subsp. *nikolai*. Four of the morphological characters tested were similar to those of *S*. *caucasica* subsp. *nikolai*, whereas 13 were more similar to those of *S*. *lingua*. According to Thompson *et al*. [80], dominance in F1 hybrids tends to be normal, where each hybrid individual’s traits fall between the midpoint of the parental traits and the traits of one of the parents. This pattern is likely influenced by the contribution of multiple quantitative trait loci (QTL).

Our findings based on SNP data imply that most of the *S*. *× ochyrae* samples are the result of introgression. This is shown by the STRUCTURE analysis, where there is only one sample with an admixture proportion of 68% and 32% for *S*. *magnifica* and *S*. *caucasica* subsp. *nikolai* respectively, while the admixture proportions of the other four samples were around 80% and 20%. According to Baiakhmetov *et al*. [81], *S*. *caucasica* and *S*. *magnifica* emerged around 0.91 and 2.04 mya respectively and the split of *S*. *magnifica* and *S*. *caucasica* based on our evolutionary scenario falls into that range. According to Arnold [82, 83] and Mallet [77], the occurrence of backcross hybrids is typical in plant species that spontaneously hybridize. Similar findings have been observed, including *Spartina alterniflora* and *S*. *foliosa* [84], as well as some backcross hybrids within the *Stipa capillata* complex [53]. Based on a study of hybridization in *Brassica napus-Hirschfeldia incana* and *B*. *napus-Raphanus raphanistrum* [85, 86], the reproductive output can drop or even rise with each consecutive backcross event. Although in this work we did not study hybrid fertility, as shown in our previous studies, *Stipa* hybrids had a lower pollen viability of 20–60% compared to their parents, as shown in *S*. × *fallax*, *S*. × *heptapotamica*, and *S*. × *tadzhikistanica* M. Nobis [10, 46, 87]. Viability reduction in hybrids may be caused by disturbed meiosis during micro- and/or microsporogenesis [88–90].

Reproductive barriers contribute to reproductive isolation (RI) by limiting gene flow across species when compared to within-species crosses [91]. Successful hybridization and introgression without such constraints could put the parent species at risk through genetic assimilation or population displacement [92]. In sympatric populations that are quite common in *Stipa* species, we suppose that hybridization is frequent, and it could cause a large number of cryptic species [10]. The absence of strong reproductive barriers allows gene flow between species, whereas vegetative propagation helps these species survive in sympatric populations [93]. Climate change can also impact pre- and post-reproductive barriers, such as temporal and geographical isolation [94, 95]. These barriers are crucial for affecting local adaptation and maintaining the genetic diversity of the species [94], while also providing hybrids with benefits in inhabiting certain niches relative to their parent species [96].

In this paper, we provide the first evidence that *S*. *caucasica* subsp. *nikolai* rather than *S*. *caucasica* subsp. *caucasica* was involved in this hybridization process in the mountains of Central Asia. The hybridization time scale is estimated to have occurred around 0.036–0.012 mya in the case of *S*. *caucasica* subsp. *nikolai* and *S*. *lingua* (S8 Table). Meanwhile, the hybridization time scale *S*. *caucasica* subsp. *nikolai* and *S*. *magnifica* occurred around 0.097–0.032 mya followed by introgression around 0.034–0.011 mya (S7 Table). These two processes occurred in the late Pleistocene up to the early Holocene. Climate fluctuations throughout the Pleistocene epoch had a significant impact on the emergence of *Stipa* and a number of other neoendemic species [57]. Both hybrid taxa origins are consistent with previous studies [53, 57, 68, 69] of *Stipa*. Central Asia experienced a wetter climate during the early and late Holocene, while drier conditions prevailed during the Middle Holocene [97, 98]. Suitable climatic conditions and high vegetation cover dominated by Poaceae make it possible for hybridization and introgression among *Stipa* populations. According to Nobis *et al*. [40], *S*. *caucasica* s. lato has a relatively wide distribution range, covering Tian Shan, Pamir, Alai, Turkestan, and Karakorum mountains adapted to middle-high mountain elevations (from 1,000 to 3,600 m). Based on its distribution, *S*. *caucasica* s. lato likely possesses a broad spectrum of tolerance to temperature and precipitation. According to Djamali *et al*. [99], there are two types of climate in the Pamir-Alai Mountains: a wet continental climate in the northern Pamir-Alai and Tian Shan regions and a dry Irano-Turanian climate in the western part of Pamir-Alai. The extensive range of *S*. *caucasica* s. lato increases the probability of hybridization with species such as *S*. *magnifica* and *S*. *lingua*, which both are rather adapted to dry and warm climates and lower (middle) mountain elevations (from 600 to 2,500 m; [40]). Thus, currently observed climate warming may favor the spreading of species to higher elevations [57, 100], resulting in distribution extension and an increase in the number of hybridization events between the aforementioned taxa and *S*. *caucasica* s. lato (or even with other *Stipa* taxa) on the mid-to-upper mountain elevations.

It has been suggested that a pool of alleles with the capacity for adaptation is essential for the fast diversification and/or shifts of lineages into a wide variety of habitats. In many cases, adaptable alleles were often transferred across species borders via hybridization and introgression [101]. The genetic variety needed for adaptation to new environments may arise from adaptive loci gained through hybridization, which might be a significant source of genetic variation. Recent hybridization shows how adaptive loci can move between parental species and/or hybrids, possibly encouraging diversification [102]. Recent studies on hybridization processes have also found that one of the two ancestral lineages frequently contributed disproportionately to putative adaptive loci. According to Taylor *et al*. [103], adaptive loci obtained through hybridization might be a significant source of genetic diversity for adaptation to a new environment. Furthermore, different candidate loci may assist a hybrid to become reproductively isolated from its parents. For instance, hybrid speciation in *Ostryopsis* (Betulaceae) led to a stable hybrid that became reproductively isolated from one parent due to adaptation to iron-rich soils and from the other parent via loci associated with flowering time [104].

In conclusion, an integrated taxonomy approach has provided important information on hybridization and backcrossing events involving *S*. *caucasica* s. lato, *S*. *magnifica*, and *S*. *lingua*. Genetic data and the appearance of intermediate and extreme morphological traits help delimit and distinguish natural hybrids from the pure species. These findings advance our knowledge of the dynamics of evolution and speciation in *Stipa* and emphasize the significance of taking into account both morphological and genetic information when researching complex organisms. Climate change can cause changes in distribution ranges of many plant species. It could impact hybridization and backcrossing frequency in species that up to date were geographically isolated. This issue will be a challenge for our further studies on the genus *Stipa*.

### Identification key for species of *Stipa* sect. *Smirnovia* Tzvel. having unigeniculate and throughout pilose awns

The analyzed group comprises taxa with having unigeniculate awns with columns (lower segment of the awn) covered by hairs (0.2–)0.5–2.5(–4.5) mm long and falcate, flexuous or straight and setae (upper segment of the awn) covered by 0.6–11 mm long hairs.

1. Awn unigeniculate………………………… **2**
• Awn uni- or sometimes indistinctly bigeniculate …………………………**16**
2. Seta (the upper segment of the awn) straight, (4.4–)5.5–12.5(–14.0) times longer than column (the lower segment of the awn) ………………………… **3**
• Seta falcate, arcuate, flexuous or straight, (1.6–)2.3–4.2(–5.2) times longer than column…………………………**10**
3. Glumes 14–19 mm long, flower (anthecium) 6.3–8.5 mm long, densely hairy throughout, column 4–7 mm long covered by 1.6–3 mm long hairs, seta 45–65 mm long covered by 2.5–4.2 mm long hairs ………………………… ***S*. *mongolorum*** Tzvel.
• Glumes over 35 mm long, flower (anthecium) over 9 mm long covered with hairs arranged in 7 lines, column over 9 mm long, seta covered by hairs over 4 mm long ………………………… **4**
4. Glumes 35–45 mm long, anthecium 9.5–11.2(–11.5) mm long, seta with hairs (3–)3.8–5.5(–6) mm long ………………………… ***S*. *aktauensis*** Roshev.
• Glumes (41–)44–90(–96) mm long, anthecium (11.0–)12–16(–16.5) mm long, seta with hairs (5.5–)6–9.8(–11.5) mm long ………………………… **5**
5. Leaves distinctly scabrous at the abaxial surface due to spinules and prickles, callus base narrowly cuneate (not expanded) ca. 0.2 mm wide, lemma without or with poorly developed ring of unequal hairs at the apex………………………… ***S*. *narynica*** M. Nobis
• Leaves glabrous and smooth at the abaxial surface, callus base broadly cuneate to ovate, lemma with well-developed ring of long hairs at the apex………………………… **6**
6. Glumes 44–55 mm long, callus base broadly cuneate 0.2–0.3 mm wide, adaxial surface of leaves with hairs 0.1–0.15 mm long, seta 4.4–5.0 times longer than column…………………………***S*. × *ochyrae*** M. Nobis & Klichowska
• Glumes 60–98 mm long, callus base expanded to 0.35–0.75 mm wide………………………… **7**
7. Seta 4.5–6.0 times longer than column, column densely covered by (0.7–)1.0–1.5(–2.0) mm long hairs, callus base broadly cuneate 0.35–0.5 mm wide, adaxial surface of leaves covered by 0.1–0.25 mm long hairs………………………… ***S*. *× muksuensis*** M. Nobis, Klichowska, A. Nowak, P. Sinaga
• Seta 5.5–14.0 times longer than column, column densely covered by 2.0–3.6 mm long hairs, callus expanded to a wide 0.45–0.75 mm………………………… **8**
8. Leaves of vegetative shoots flat or convolute, at the adaxial surface densely and very shortly pilose, with hairs 0.05–0.10(–0.15) mm long, ligules of the vegetative shoots very densely pilose with hairs up to 3 mm long, base of the callus pyriform………………………… ***S*. *magnifica*** A. Junge
• Leaves of vegetative shoots convolute, at the adaxial surface densely covered by 0.2–0.55 mm long hairs, ligules of the vegetative shoots pilose with up to 2 mm long hairs, base of the callus ovate or broadly cuneate………………………… **9**
9. Dorsal part of callus glabrous or shortly pilose only at the base of lemma, ventral part of callus more or less densely pilose, base of the callus ovate, goblet-like concave distinctly expanded to a wide 0.55–0.75 mm, lower segment of awn sparsely pilose, at the adaxial surface of leaves densely covered by 0.2–0.4 mm long hairs………………………… ***S*. *lingua*** A. Junge
• Callus densely pilose all-around, base of the callus broadly cuneate to a wide 0.45–0.60 mm, lower segment of awn densely pilose, at the adaxial surface of leaves densely covered by 0.35–0.55 mm long hairs………………………… ***S*. *ovczinnikovii*** Roshev.
10. Callus 0.8–1.2(–1.5) mm long, hairs in the dorsal part of the callus strongly falcate ………………………… ***S*. *drobovii*** (Tzvel.) Czerep.
• Callus (1.6–)1.8–3.5(–4) mm long, hairs in the dorsal part of the callus straight or flexuous…………………………**11**
11. The longest ligules of the vegetative shoots up to 0.2 mm long, glumes 34–61 mm long …………………………**12**
• The longest ligules of the vegetative shoots 0.3–2.5 mm long, if ligules shorter (up to 0.2 mm) than glumes 18–28 mm long…………………………**15**
12. Adaxial surface of leaves of vegetative shoots covered with hairs 0.15–0.6 mm long…………………………**13**
• Adaxial surface of leaves of vegetative shoots covered with prickle-hairs 0.08–0.1 mm long …………………………**14**
13. Dorsal part of the callus with hairs 1.5–2 mm long and flexuous, callus (1.5–)1.6–2.1(–2.3) mm long, abaxial surface of vegetative leaves glabrous or pubescent………………………… ***S*. *× subdrobovii*** M. Nobis & A. Nowak
• Dorsal part of the callus with hairs 0.2–0.5 mm long and straight, callus 2–2.4 mm long, abaxial surface of vegetative leaves glabrous and smooth ………………………… ***S*. *caucasica*** Schmalh. var. ***fanica*** M. Nobis, P.D. Gudkova & A. Nowak
14. The longest hairs in the lower (1/3–1/2) part of the lower segment of awn (column) 0.2–0.7(–0.9) mm long ………………………… ***S*. *caucasica*** Schmalh. subsp. ***nikolai*** M. Nobis, A. Nobis & A. Nowak
• The longest hairs in the lower (1/3–1/2) part of the lower segment of awn (column) (0.9–)1–2(–2.5) mm long ………………………… ***S*. *caucasica*** Schmalh. subsp. ***caucasica***
15. The longest ligules up to 0.3(–0.6) mm long, awn unigeniculate, column with hairs (0.8–)1.0–2.3(–3.0) mm long, glumes 19–28 mm long………………………… ***S*. *glareosa*** P.A. Smirn.
• The longest ligules 0.5–2.3 mm long, awn indistinctly bigeniculate, column with hairs 0.1–1.0 mm long, glumes 18–61 mm long………………………… **16**
16. Glumes (50–)55–61 mm long, awn (10.9–)13.2–16.4(–17.6) cm long, leaves of vegetative shoots densely pubescent………………………… ***S*. × *fallax*** M. Nobis & A. Nowak
• Glumes up to 45 mm long, awn 5–13.5(–16) cm long, leaves of vegetative shoots glabrous or scabrous, rarely densely pubescent ………………………… **17**
17. Seta with hairs ≤1 mm long ………………………… **18**
• Seta with hairs ≥2.5 mm long ………………………… **19**
18. Awns (11–)13–16 cm long, glumes 32–40 mm long, vegetative leaves glabrous or distinctly scabrous due to densely distributed spinules and prickles ………………………… ***S*. *× balkanabatica*** M. Nobis & P.D Gudkova
• Awns 7–11 cm long, glumes 24–30 mm long, vegetative leaves glabrous or slightly scabrous due to scattered prickles ………………………… ***S*. *× consanguinea*** Trin. & Rupr.
19. Glumes 35–43(–45) mm long, awn 11.5–14 cm long, column with 0.1–0.3 mm long hairs, leaves of vegetative shoots glabrous…………………………***S*. *× gnezdilloi*** Pazij
• Glumes 18–38 mm long, awn up to 11 cm long, column with 0.3–1.0 mm long hairs, leaves of vegetative shoots scabrous or glabrous…………………………**20**
20. Glumes 18–26 mm long, column with hairs 0.5–1 mm long, leaves scabrous to slightly scabrous………………………… ***S*. *× albasiensis*** L.Q. Zhang &K. Guo
• Glumes 26–38 mm long, column with hairs 0.3–0.6 mm long, leaves glabrous to slightly scabrous………………………… ***S*. *× tzvelevii*** Ikonn.

## Supporting information

S1 Table List of examined samples. (DOCX)

S2 Table Results of the Principal Component Analysis (PCA) and Factor Analysis on Mixed Data (FAMD). PCA was based on 11 morphological characters and FAMD was based on 14 morphological characters of 11 *Stipa* taxa having unigeniculate awns (qualitative characters indicated by *). We display the factor loadings on the first three principal components (for PCA)/dimensions (for FAMD) and results of one-way ANOVA (p<0.05).
(DOCX)

S3 Table PCA results of *Stipa magnifica*, *S*. *caucasica* subsp. *caucasica*, *S*. *caucasica* subsp. *nikolai* and putative hybrid of *S*. *magnifica* × *S*. *caucasica*. Results of the principal component analysis (PCA) for the specimens as OTUs based on 29 morphological characters. We display the factor loadings on the first three principal components and results of one-way ANOVA (p<0.05): F and p values.
(DOCX)

S4 Table PCA results of *Stipa lingua*, *S*. *caucasica* subsp. *caucasica*, *S*. *caucasica* subsp. *nikolai* and putative hybrid of *S*. *lingua* × *S*. *caucasica*. Results of the Principal Component Analysis (PCA) for the specimens as OTUs based on 29 morphological characters. We display the factor loadings on the first three principal components and results of one-way ANOVA (p<0.05): F and p values.
(DOCX)

S5 Table Morphological character expression for hybrid *S*. *magnifica* × *S*. *caucasica* subsp. *nikolai* and cooccurring parental taxa. Measurements are given in millimeters (mm). F values are generated using ANOVA if normality assumptions are met or H values is generated using Kruskal-Wallis test if normality assumptions are not met. Note: *magnifica*-like (hybrid does not differ significantly from *S*. *magnifica*), *nikolai*-like (hybrid does not differ significantly from *S*. *caucasica* subsp. *nikolai*), n.s. (no significant differences between groups, p-value<0.05).
(DOCX)

S6 Table Morphological character expression for hybrid *S*. *lingua* × *S*. *caucasica* subsp. *nikolai* and cooccurring parental taxa. Measurements are given in millimeters (mm). F values are generated using ANOVA if normality assumptions are met or H values is generated using Kruskal-Wallis test if normality assumptions are not met. Note: *lingua*-like (hybrid does not differ significantly from *S*. *lingua*), *nikolai*-like (hybrid does not differ significantly from *S*. *caucasica* subsp. *nikolai*), n.s. (no significant differences between groups, p-value<0.05).
(DOCX)

S7 Table Parameter estimation for chosen scenario of *S*. *magnifica*, *S*. *caucasica* and their putative hybrid based on DIYABC. Note: N is the number of estimated population size, t is time (generations) and set within the range 10–500000, r is population admixture rate.
(DOCX)

S8 Table Parameter estimation for chosen scenario for *S*. *lingua*, *S*. *caucasica* and their putative hybrid based on DIYABC. Note: N is the number of estimated population size, t is time (generations) and set within the range 10–500000, r is population admixture rate.
(DOCX)

S1 Fig Box plots of 18 quantitative characters for *S*. *caucasica* subsp. *caucasica*, *S*. *caucasica* subsp. *nikolai*, *S*. *magnifica*, *S*. *lingua*, *S*. *magnifica* × *S*. *caucasica*, *S*. *lingua* × *S*. *caucasica*. Characters were measured in millimeters (mm).
(PDF)

S2 Fig Linear Discriminant Analysis plot of scenario 1 and 2 of the occurrence of *S*. *magnifica* × *S*. *caucasica*. These two scenarios are involving only hybridization of *S*. *magnifica* and *S*. *caucasica* subsp. *nikolai* or *S*. *magnifica* and *S*. *caucasica* subsp. *caucasica*.
(PDF)

S3 Fig Linear Discriminant Analysis plot of scenario 3 and 4 of the occurrence of *S*. *magnifica* × *S*. *caucasica*. These two scenarios are involving hybridization of *S*. *magnifica* and *S*. *caucasica* subsp. *nikolai* and backcross to *S*. *magnifica*.
(PDF)

S4 Fig Linear Discriminant Analysis plot of scenario 5 and 6 of the occurrence of *S*. *lingua* × *S*. *caucasica*. These two scenarios are involving only hybridization of *S*. *lingua* with *S*. *caucasica* subsp. *nikolai* or *S*. *lingua* with *S*. *caucasica* subsp. *caucasica*.
(PDF)

S5 Fig Holotype of Stipa *× muksuensis* M. Nobis, Klichowska, A. Nowak, P. Sinaga. (PDF)

S6 Fig Holotype of *Stipa × ochyrae* M. Nobis, Klichowska. (PDF)
