# Supplementary material for: Hybridization and introgression events in cooccurring populations of closely related grasses (Poaceae: Stipa) in high mountain steppes of Central Asia
Source: PLoS One. 2024 Feb 27;19(2):e0298760. doi: 10.1371/journal.pone.0298760 (PMC10898772; doi:10.1371/journal.pone.0298760)
Supplement: S1 Table — (DOCX) [file pone.0298760.s001.docx]

**S1 Table.** **List of examined samples**

| **Species** | **Tag No.** | **Country** | **Location** | **Coordinate** | | **Herbarium** |
| --- | --- | --- | --- | --- | --- | --- |
| *S caucasica subsp. caucasica* | caucS/53/7 | Kazakhstan | 6 km SE of Tastybastau settl. (160 km NE of Almaty) | 44°14'32.54"N | 78°29'55.77"E | KRA |
| *S caucasica subsp. caucasica* | caucS/52/72 | Kyrgyzstan | Kara-Balta River valley, to the N of tunnel | 42°27'50.50"N | 73°49'25.46"E | KRA |
| *S caucasica subsp. caucasica* | caucS/53/3 | Kyrgyzstan | 25 km N from the village of Sosnovka (before the pass) |  |  | KRA |
| *S caucasica subsp. caucasica* | caucSt87 | Kyrgyzstan | right solpe of the Chu river valley, ca. 2 km N of Krasnyi Most settlement, SE of Dzhel-Aryk | 42°37'5.33"N | 75°49'30.16"E | KRA |
| *S caucasica subsp. caucasica* | cauc1251/24 | Kazakhstan | ca. 92 km S of Taldykorgan, ca. 163 km NE of Almaty | 44°11'23.26"N | 78°34'26.57"E | KRA |
| *S caucasica subsp. caucasica* | caucSt85 | Kazakhstan | 2.5 km NNE of Karlygash settlement (100 km NE of Almaty) | 44°13'41.86"N | 77°42'30.67"E | KRA |
| *Stipa caucasica* subsp. *nikolai* | nikoS/51/32b | Kyrgyzstan | at the edge of the Tien Shan Mountains, Naryn River Valley, ca. 44 km to SW from Toktogul Reservoir, ca. 22 km to the NE from Tash-Kumyr city | 41°30'8.73"N | 72°23'21.51"E | KRA |
| *Stipa caucasica* subsp. *nikolai* | nikoSt113 | Tajikistan | on hills in Kyzyl-Suu River Valley, Kashat settl. near Karakendzhe | 39°18'18.96"N | 71°27'23.80"E | KRA |
| *Stipa caucasica* subsp. *nikolai* | nikoS/53/33 | Tajikistan | Zeravshan Mts., high mountain steppe, between stones, on the left slope of Iskanderdarya river valley, ca. 0.5 km E of Serimadarum lake (near Iskanderkul Lake) | 39°05'08"N | 68°22'46"E | KRA |
| *Stipa caucasica* subsp. *nikolai* | nikoSI/5 | Tajikistan | Kshtut River Valley | 39°13'43.24"N | 68° 1'25.48"E | KRA |
| *Stipa caucasica* subsp. *nikolai* | nikoSt86 | Tajikistan | on the left slope of Mogendarya river valley, ca. 5 km NEE of Khurmi settlement, near Mogien | 39°16'29"N | 67°36'10"E | KRA |
| *Stipa caucasica* subsp. *nikolai* | nikoSt83 | Tajikistan | Kshtut River Valley ca. 3 km S of Gaza | 39°16'36''N | 68°02'59"E | KRA |
| *Stipa drobovii* var. *drobovii* | drobS/11/18 | Tajikistan | near the village of Khudgif Bolo, E of Yarm (about 130 km E of Aini) | 39°27'28''N | 70°02'54"E | KRA |
| *Stipa drobovii* var. *drobovii* | drobSt115 | Tajikistan | among stones on left slope of the Iskanderdarya River Valley, ca. 0.5 km E of Serimadarum Lake (near Iskanderkul Lake) | 39°05'N | 68°23'E | KRA |
| *Stipa drobovii* var. *iskanderkulica* | iskaSt82 | Tajikistan | Kshtut River Valley ca. 3 km S of Gaza | 39°16'36''N | 68°02'59"E | KRA |
| *Stipa x muksuensis* | li×caSt68 | Tajikistan | on the left slope of Muksu river valley, Sartala settl. Near Karakendzhe (Kyzyl-Suu river valley) | 39°14'58.01"N | 71°26'14.03"E | KRA |
| *Stipa x muksuensis* | li×caSt69 | Tajikistan | on the left slope of Muksu river valley, Sartala settl. Near Karakendzhe (Kyzyl-Suu river valley) | 39°14'58.01"N | 71°26'14.03"E | KRA |
| *Stipa x muksuensis* | li×caSt70 | Tajikistan | on the left slope of Muksu river valley, Sartala settl. Near Karakendzhe (Kyzyl-Suu river valley) | 39°14'58.01"N | 71°26'14.03"E | KRA |
| *Stipa x ochyrae* | ma×caSt75 | Kyrgysztan | near the road, in the E part of Tash-Koroo settl., ca. 9 km SW of Gulcha | 40°15'08"N | 73°23'08"E | KRA |
| *Stipa x ochyrae* | ma×caSt111 | Kyrgysztan | near the road, in the E part of Tash-Koroo settl., ca. 9 km SW of Gulcha | 40°15'08"N | 73°23'08"E | KRA |
| *Stipa x ochyrae* | ma×caSt71 | Kyrgysztan | near the road, in the E part of Tash-Koroo settl., ca. 9 km SW of Gulcha | 40°15'08"N | 73°23'08"E | KRA |
| *Stipa x ochyrae* | ma×caSt74 | Kyrgysztan | ca. 57 km to the SE from Osh city, between Gülczö and Tashkoro villages, near the road M41 | 40°15'5.72"N | 73°23'5.15"E | KRA |
| *Stipa x ochyrae* | ma×caSt110 | Kyrgysztan | ca. 57 km to the SE from Osh city, between Gülczö and Tashkoro villages, near the road M41 | 40°15'5.72"N | 73°23'5.15"E | KRA |
| *Stipa narynica* | narySt61 | Kyrgyzstan | on outcroppings of limestone rocks on mountain slopes, 2.5 km E from Tashkumyr | 41°19'42.98"N | 72°13'28.07"E | KRA |
| *Stipa narynica* | narySt81 | Kyrgyzstan | on outcroppings of limestone rocks on mountain slopes, 2.5 km E from Tashkumyr | 41°19'42.98"N | 72°13'28.07"E | KRA |
| *Stipa lingua* | lingSt67 | Tajikistan | near the botanical garden in Horog | 37°28'28"N | 71°36'07"E | KRA |
| *Stipa lingua* | lingSt66 | Tajikistan | on the left slope of Muksu river valley, Sartala settl. Near Karakendzhe (Kyzyl-Suu river valley) | 39°14'58.01"N | 71°26'14.03"E | KRA |
| *Stipa lingua* | lingszSt652 | Tajikistan | on the left slope pf Muksu river valley, Sartala settl. Near Karakendzhe (Kyzyl-Suu river valley) | 39°14'58.01"N | 71°26'14.03"E | KRA |
| *Stipa magnifica* | magnSt76 | Kyrgyzstan | slope, in valley near the road, 10 km S of Kara-Echki (SE of Osh) | 40°26'1.32"N | 73°5'1.34"E | KRA |
| *Stipa magnifica* | magnSt78 | Kyrgyzstan | near the road, in the E part of Tash-Koroo settl., ca. 9 km SW of Gulcha | 40°15'08"N | 73°23'08"E | KRA |
| *Stipa magnifica* | magnSt79 | Kyrgyzstan | 9.5 km S of Kara-Akdzhar (left slope valley) | 40°26'4.63"N | 73°4'58,33"E | KRA |
| *Stipa magnifica* | magnSt80 | Kyrgyzstan | near the road, in the E part of Tash-Koroo settl., ca. 9 km SW of Gulcha | 40°15'08"N | 73°23'08"E | KRA |
| *Stipa ovczinnikovii* | ovcziSt62 | Tajikistan | ca. 24 km of Aini, ca. 90 km ESE of Panjakent | 39° 10' 45.7788" N | 39° 10' 45.7788" N | KRA |
| *Stipa ovczinnikovii* | ovcziSt63 | Tajikistan | ca. 24 km of Aini, ca. 90 km ESE of Panjakent | 39° 10' 45.7788" N | 39° 10' 45.7788" N | KRA |
| *Stipa ovczinnikovii* | ovcziSt64 | Tajikistan | ca. 24 km of Aini, ca. 90 km ESE of Panjakent | 39° 10' 45.7788" N | 39° 10' 45.7788" N | KRA |
| *Stipa glareosa* | glarS/49/74 | Mongolia | in 6 km NW of Dzun Gobi Somona |  |  | KRA |
| *Stipa glareosa* | glarS/49/75 | Mongolia | in 30 km NW of Dzun Gobi Somona |  |  | KRA |
