# Supplementary material for: Hybridization and introgression events in cooccurring populations of closely related grasses (Poaceae: Stipa) in high mountain steppes of Central Asia
Source: PLoS One. 2024 Feb 27;19(2):e0298760. doi: 10.1371/journal.pone.0298760 (PMC10898772; doi:10.1371/journal.pone.0298760)
Supplement: S2 Table — PCA was based on 11 morphological characters and FAMD was based on 14 morphological characters of 11 Stipa taxa having unigeniculate awns (qualitative characters indicated by *). We display the factor loadings on the first three principal components (for PCA)/dimensions (for FAMD) and results of one-way ANOVA (p<0.05). (DOCX) [file pone.0298760.s002.docx]

**S2 Table. Results of the Principal Component Analysis (PCA) and Factor Analysis on Mixed Data (FAMD).** PCA was based on 11 morphological characters and FAMD was based on 14 morphological characters of 11 *Stipa* taxa having unigeniculate awns (qualitative characters indicated by *). We display the factor loadings on the first three principal components (for PCA)/dimensions (for FAMD) and results of one-way ANOVA (p<0.05).

| **Character** | **PCA** | | | **FAMD** | | | **p value** |
| --- | --- | --- | --- | --- | --- | --- | --- |
|  | **PC1** | **PC2** | **PC3** | **Dim.1** | **Dim.2** | **Dim.3** |  |
| Floret (=anthecium) length | **0.908** | -0.159 | 0.037 | **0.78** | 0.03 | 0.02 | <0.05 |
| Callus length | 0.468 | **-0.782** | 0.141 | 0.18 | **0.62** | 0.02 | <0.05 |
| Awn length | **0.959** | -0.100 | 0.119 | **0.9** | 0.02 | 0.04 | <0.05 |
| Callus’ foot ring width | **0.661** | 0.435 | -0.519 | 0.51 | 0.16 | 0.21 | <0.05 |
| Seta length | **0.962** | -0.054 | 0.150 | **0.91** | 0.01 | 0.05 | <0.05 |
| Ratio of seta and column | **0.855** | 0.103 | 0.365 | **0.71** | 0.01 | 0.13 | <0.05 |
| Length of hair on column | 0.557 | **0.671** | 0.337 | 0.32 | 0.4 | 0.06 | <0.05 |
| Ratio of length: seta hair to column hair | -0.153 | **-0.864** | -0.257 | 0.03 | **0.7** | 0.03 | <0.05 |
| Lower glume length | **0.922** | -0.025 | -0.315 | **0.84** | 0 | 0.03 | <0.05 |
| Length of hairs on adaxial surface of vegetative leaf | -0.204 | **0.866** | -0.162 | 0.03 | **0.77** | 0.02 | <0.05 |
| Upper glume length | **0.917** | -0.026 | -0.323 | **0.83** | 0 | 0.04 | <0.05 |
| Character of abaxial surface of vegetative leaves * |  |  |  | 0.05 | 0.26 | 0.22 | <0.05 |
| Character of dorsal surface of the callus * |  |  |  | 0.22 | 0.03 | 0.33 | <0.05 |
| Character of callus’ foot ring * |  |  |  | 0.58 | 0.03 | 0 | <0.05 |
