# Supplementary material for: Hybridization and introgression events in cooccurring populations of closely related grasses (Poaceae: Stipa) in high mountain steppes of Central Asia
Source: PLoS One. 2024 Feb 27;19(2):e0298760. doi: 10.1371/journal.pone.0298760 (PMC10898772; doi:10.1371/journal.pone.0298760)
Supplement: S3 Table — Results of the principal component analysis (PCA) for the specimens as OTUs based on 29 morphological characters. We display the factor loadings on the first three principal components and results of one-way ANOVA (p<0.05): F and p values. (DOCX) [file pone.0298760.s003.docx]

**S3 Table. PCA results of *Stipa magnifica*, S. caucasica subsp. *caucasica*, *S. caucasica* subsp. *nikolai* and putative hybrid of *S. magnifica* × *S. caucasica*.** Results of the Principal Component Analysis (PCA) for the specimens as OTUs based on 29 morphological characters. We display the factor loadings on the first three principal components and results of one-way ANOVA (p<0.05): F and p values.

| **Character** | **Factor loadings** | | | **ANOVA** | |
| --- | --- | --- | --- | --- | --- |
|  | **PC1** | **PC2** | **PC3** | **F value** | **p value** |
| Floret (=anthecium) length | **-0.880** | -0.109 | -0.136 | 95.160 | <0.05 |
| Callus length | -0.073 | -0.059 | -0.113 | 2.331 | 0.076 |
| Dorsal hair length on callus | -0.250 | -0.197 | **0.627** | 15.410 | <0.05 |
| Ventral hair length on callus | -0.269 | 0.114 | 0.532 | 23.910 | <0.05 |
| Awn length | **-0.966** | 0.056 | -0.095 | 534.800 | <0.05 |
| Callus’ foot ring width | **-0.869** | 0.121 | 0.037 | 395.500 | <0.05 |
| Corolla hair length | **-0.771** | 0.244 | 0.105 | 153.700 | <0.05 |
| Distance from the end of the dorsal line of hairs to the top of the lemma | 0.079 | -0.125 | -0.497 | 27.200 | <0.05 |
| Distance from the end of the dorsal line of hairs to the top of the lemma | 0.086 | -0.011 | -0.128 | 2.855 | <0.05 |
| Column (lower segment of the awn) length | -0.290 | -0.564 | -0.242 | 0.538 | 0.657 |
| Seta (upper segment of the awn) length | **-0.964** | 0.100 | -0.078 | 714.600 | <0.05 |
| Ratio of seta length to column length | **-0.904** | 0.251 | -0.027 | 585.500 | <0.05 |
| Length of hair on column | **-0.835** | 0.346 | 0.009 | 225.600 | <0.05 |
| Length of hair on seta | **-0.831** | 0.159 | -0.166 | 124.300 | <0.05 |
| Ratio of length: seta hair to column hair | 0.598 | -0.132 | -0.421 | 97.460 | <0.05 |
| Lower glume length | **-0.909** | -0.038 | -0.126 | 141.400 | <0.05 |
| Length of hairs on adaxial surface of vegetative leaf | -0.147 | 0.189 | -0.114 | 1.964 | 0.121 |
| Vegetative leaves length | -0.335 | **-0.633** | -0.375 | 4.631 | <0.05 |
| Vegetative leaves width | **-0.622** | -0.543 | -0.027 | 27.380 | <0.05 |
| Column width | 0.042 | **-0.644** | 0.259 | 8.055 | <0.05 |
| Callus’ foot ring length | 0.342 | -0.215 | -0.131 | 26.700 | <0.05 |
| Floret (=anthecium) width | -0.593 | -0.221 | 0.226 | 31.890 | <0.05 |
| Length of dorsal hairs on lemma | -0.316 | -0.496 | 0.235 | 4.120 | <0.05 |
| Length of ventral hairs on lemma | **-0.719** | -0.189 | 0.364 | 67.410 | <0.05 |
| Culm length | -0.566 | -0.283 | -0.423 | 20.150 | <0.05 |
| Length of ligule of vegetative leaves | 0.154 | 0.298 | -0.392 | 3.910 | <0.05 |
| Upper glume length | **-0.891** | -0.060 | -0.155 | 120.200 | <0.05 |
| Length of hairs on ligule of vegetative shoots | -0.112 | 0.101 | -0.421 | 77.570 | <0.05 |
| Upper culm’s sheath width | **0.665** | -0.344 | 0.094 | 86.970 | <0.05 |
