# Supplementary material for: Hybridization and introgression events in cooccurring populations of closely related grasses (Poaceae: Stipa) in high mountain steppes of Central Asia
Source: PLoS One. 2024 Feb 27;19(2):e0298760. doi: 10.1371/journal.pone.0298760 (PMC10898772; doi:10.1371/journal.pone.0298760)
Supplement: S5 Table — Measurements are given in millimeters (mm). F values are generated using ANOVA if normality assumptions are met or H values is generated using Kruskal-Wallis test if normality assumptions are not met. Note: magnifica-like (hybrid does not differ significantly from S. magnifica), nikolai-like (hybrid does not differ significantly from S. caucasica subsp. nikolai), n.s. (no significant differences between groups, p-value<0.05). (DOCX) [file pone.0298760.s005.docx]

**S5 Table**. **Morphological character expression for hybrid *S. magnifica* × *S. caucasica* subsp. *nikolai* and cooccurring parental taxa*.*** Measurements are given in millimeters (mm). F values are generated using ANOVA if normality assumptions are met or H values is generated using Kruskal-Wallis test if normality assumptions are not met. Note: *magnifica*-like (hybrid does not differ significantly from *S. magnifica*), *nikolai*-like (hybrid does not differ significantly from *S. caucasica* subsp. *nikolai*), n.s. (no significant differences between groups, p-value<0.05).

| Character | *S. magnifica* | *S. magnifica* × *S. caucasica* | *S. caucasica* subsp*. nikolai* | Hybrid character | F value |
| --- | --- | --- | --- | --- | --- |
| Floret (=anthecium) length | 14.518±0.24a | 12.653±0.10a | 10.802±0.10b | *magnifica*-like | 77.69 |
| Callus length | 2.109±0.05a | 2.300±0.06a | 2.165±0.02a | n.s. | 5.399 |
| Dorsal hair length on callus | 1.214±0.05a | 1.193±0.06ab | 1.065±0.02b | codominance | 6.551 |
| Ventral hair length on callus | 1.636±0.06b | 1.867±0.07a | 1.481±0.02c | positive transgressive | 26.31 |
| Awn length | 254.227±5.90a | 151.600±2.49b | 95.088±1.53c | intermediate | 734.6 |
| Callus’ foot ring width | 0.502a | 0.260±0.01b | 0.23±0b | *nikolai*-like | 58.98 |
| Corolla hair length | 1.645±0.06a | 1.660±0.08a | 0.673±0.02b | *magnifica*-like | 84.08 |
| Distance from the end of the dorsal line of hairs to the top of the lemma | 2.786±0.30a | 1.160±0.12b | 2.744±0.06a | negative transgressive | 32.55 |
| Distance from the end of the ventral line of hairs to the top of the lemma | 1.182±0.10a | 0.580±0.10b | 1.166±0.08a | negative transgressive | 7.993 |
| Column (lower segment of the awn) length | 25.273±0.73a | 24.433±0.32a | 24.083±0.38a | n.s. | 2.213 |
| Seta (upper segment of the awn) length | 228.955±5.58a | 127.167±2.23b | 71.004±1.29c | intermediate | 940.2 |
| Ratio of seta length to column length | 9.181±0.30a | 5.204±0.06a | 2.983±0.05b | intermediate | 85.78 |
| Length of hair on column | 3.745±0.34a | 2.213±0.07a | 0.524±0.02b | *magnifica*-like | 85.16 |
| Length of hair on seta | 8.400±0.15a | 7.040±0.13b | 5.462±0.06c | intermediate | 187.9 |
| Ratio of length: seta hair to column hair | 2.552±0.18a | 3.228±0.12a | 11.454±0.35b | *magnifica*-like | 84.7 |
| Lower glume length | 70.727±1.25a | 51.867±1.04a | 41.333±0.62b | *magnifica*-like | 75.23 |
| Length of hairs on adaxial surface of vegetative leaf | 0.1±0a | 0.1±0a | 0.099±0a | n.s. | 1.317 |
| Vegetative leaves length | 204.636±18.73a | 113.867±6.10b | 151.967±7.39b | *nikolai*-like | 11.67 |
| Vegetative leaves width | 1.068±0.06a | 0.667±0.03b | 0.700±0.01b | *nikolai*-like | 35.97 |
| Column width | 0.516±0.03a | 0.413±0.04a | 0.538±0.01b | *magnifica*-like | 5.496 |
| Callus’ foot ring length | 0.626±0.01a | 0.807±0.05b | 0.837±0.01b | *nikolai*-like | 45.44 |
| Floret (=anthecium) width | 1.223±0.02a | 1.020±0.02b | 0.995±0.01b | *nikolai*-like | 38.69 |
| Length of dorsal hairs on lemma | 1.214±0.04a | 1.087±0.04ab | 1.048±0.02b | codominance | 11.11 |
| Length of ventral hairs on lemma | 1.395±0.07a | 0.947±0.04b | 0.785±0.01b | intermediate | 58.58 |
| Culm length | 55.977±3.72a | 52.333±1.82a | 38.925±1.14b | *magnifica*-like | 30.93 |
| Length of ligule of vegetative leaves | 0.714±0.19a | 0.780±0.15a | 0.923±0.04b | *magnifica*-like | 8.159 |
| Upper glume length | 68.182±1.62a | 48.667±1.01a | 39.617±0.63b | intermediate | 71.2 |
| Length of hairs on ligule of vegetative shoots | 0.639±0.03a | 1.280±0.05b | 0.713±0.03a | positive transgressive | 32.08 |
| Upper culm’s sheath width | 1.289±0.04a | 2.953±0.13a | 6.605±0.16b | *magnifica*-like | 82.42 |
