# Supplementary material for: Hybridization and introgression events in cooccurring populations of closely related grasses (Poaceae: Stipa) in high mountain steppes of Central Asia
Source: PLoS One. 2024 Feb 27;19(2):e0298760. doi: 10.1371/journal.pone.0298760 (PMC10898772; doi:10.1371/journal.pone.0298760)
Supplement: S6 Table — Measurements are given in millimeters (mm). F values are generated using ANOVA if normality assumptions are met or H values is generated using Kruskal-Wallis test if normality assumptions are not met. Note: lingua-like (hybrid does not differ significantly from S. lingua), nikolai-like (hybrid does not differ significantly from S. caucasica subsp. nikolai), n.s. (no significant differences between groups, p-value<0.05). (DOCX) [file pone.0298760.s006.docx]

**S6 Table. Morphological character expression for hybrid *S. lingua* × *S. caucasica* subsp. *nikolai* and cooccurring parental taxa*.*** Measurements are given in millimeters (mm). F values are generated using ANOVA if normality assumptions are met or H values is generated using Kruskal-Wallis test if normality assumptions are not met. Note: *lingua*-like (hybrid does not differ significantly from *S. lingua*), *nikolai*-like (hybrid does not differ significantly from *S. caucasica* subsp. *nikolai*), n.s. (no significant differences between groups, p-value<0.05).

| Character | *S. lingua* | *S. lingua* × *S. caucasica* | *S. caucasica* subsp*. nikolai* | Hybrid character | F value |
| --- | --- | --- | --- | --- | --- |
| Floret (=anthecium) length | 14.095±0.23a | 13.252±0.14a | 10.802±0.10b | *lingua*-like | 8.748 |
| Callus length | 2.019±0.07a | 2.040±0.03ab | 2.165±0.02b | codominance | 7.654 |
| Dorsal hair length on callus | 0.257±0.07a | 0.944±0.05a | 1.065±0.02b | *lingua*-like | 34.82 |
| Ventral hair length on callus | 1.205±0.08a | 1.392±0.06a | 1.481±0.02b | *lingua*-like | 17.16 |
| Awn length | 186.176±4.71a | 160.720±2.85a | 95.088±1.53b | *lingua*-like | 14.49 |
| Callus’ foot ring width | 0.592±0a | 0.4±0b | 0.230±0b | *lingua*-like | 20.57 |
| Corolla hair length | 1.714±0.12a | 1.520±0.07a | 0.673±0.02b | *lingua*-like | 13.82 |
| Distance from the end of the dorsal line of hairs to the top of the lemma | 5.795±0.30a | 3.608±0.24b | 2.744±0.06b | *nikolai*-like | 8.092 |
| Distance from the end of the ventral line of hairs to the top of the lemma | 1.710±0.18a | 1.112±0.10a | 1.166±0.08a | n.s | 0.732 |
| Column (lower segment of the awn) length | 21.367±0.92a | 25.440±0.62a | 24.083±0.38a | n.s | 0.091 |
| Seta (upper segment of the awn) length | 164.810±4.45a | 135.280±2.80a | 71.004±1.29b | *lingua*-like | 15.35 |
| Ratio of seta length to column length | 8.010±0.43a | 5.407±0.20a | 2.983±0.05b | *lingua*-like | 16.56 |
| Length of hair on column | 3.419±0.23a | 1.436±0.06a | 0.524±0.02b | *lingua*-like | 14.61 |
| Length of hair on seta | 8.810±0.20a | 8.404±0.14a | 5.462±0.06b | *lingua*-like | 17.73 |
| Ratio of length: seta hair to column hair | 2.774±0.16a | 6.075±0.25b | 11.454±0.35b | *nikolai*-like | 11.66 |
| Lower glume length | 84.143±1.93a | 67.800±1.42a | 41.333±0.62b | *lingua*-like | 13.88 |
| Length of hairs on adaxial surface of vegetative leaf | 0.367±0.03a | 0.156±0.01b | 0.099±0b | *nikolai*-like | 5.38 |
| Vegetative leaves length | 127.762±13.35a | 152.840±11.67a | 151.967±7.39a | n.s. | 3.373 |
| Vegetative leaves width | 0.736±0.03a | 0.692±0.01a | 0.700±0.01a | n.s. | 5.177 |
| Column width | 0.524±0.02a | 0.596±0.02b | 0.526±0.01a | positive transgressive | 11.38 |
| Callus’ foot ring length | 0.617±0.01a | 0.968±0.01a | 0.837±0.01a | n.s | 5.352 |
| Floret (=anthecium) width | 1.095±0.03a | 1.160±0.02a | 0.995±0.01a | n.s | 3.433 |
| Length of dorsal hairs on lemma | 0.743±0.12a | 1.120±0.02ab | 1.048±0.02b | codominance | 9.715 |
| Length of ventral hairs on lemma | 1.124±0.07a | 1.032±0.01a | 0.785±0.01b | *lingua*-like | 19.5 |
| Culm length | 40.762±2.61a | 31.384±2.47a | 38.925±1.14a | n.s | 1.763 |
| Length of ligule of vegetative leaves | 0.419±0.11a | 0.420±0.01a | 0.923±0.04a | n.s | 4.929 |
| Upper glume length | 81.524±1.92a | 64.440±1.28a | 39.617±0.63b | *lingua*-like | 13.65 |
| Length of hairs on ligule of vegetative shoots | 1.140±0.01a | 0.944±0.05b | 0.713±0.03c | intermediate | 16.08 |
| Upper culm’s sheath width | 2.471±0.05b | 6.720±0.26a | 6.605±0.16a | *nikolai*-like | 4.828 |
