# Supplementary material for: Hybridization and introgression events in cooccurring populations of closely related grasses (Poaceae: Stipa) in high mountain steppes of Central Asia
Source: PLoS One. 2024 Feb 27;19(2):e0298760. doi: 10.1371/journal.pone.0298760 (PMC10898772; doi:10.1371/journal.pone.0298760)
Supplement: S7 Table — Note: N is the number of estimated population size, t is time (generations) and set within the range 10–500000, r is population admixture rate. (DOCX) [file pone.0298760.s007.docx]

**S7 Table**. **Parameter estimation for chosen scenario of *S. magnifica*, *S. caucasica* and their putative hybrid based on DIYABC.** Note: N is the number of estimated population size, t is time (generations) and set within the range 10–500000, r is population admixture rate.

|  |  |  |  |  |  | **Years (time)** | | | | |
| --- | --- | --- | --- | --- | --- | --- | --- | --- | --- | --- |
| **Parameter** | **Expectation** | **Median** | **Quantile_0.05** | **Quantile_0.95** | **Variance** | **2** | **3** | **4** | **5** | **6** |
| t1 (backcross) | 7408.01 | 5673.46 | 439.072 | 22320.8 | 4.58E+07 | 11346.92 | 17020.38 | 22693.84 | 28367.3 | 34040.76 |
| t2 (*S. magnifica* population split) | 28417.9 | 23724.1 | 4779.77 | 66809.3 | 2.36E+08 | 47448.2 | 71172.3 | 94896.4 | 118620.5 | 142344.6 |
| t3 (hybridization) | 172282 | 161864 | 78687.6 | 306384 | 1.93E+09 | 323728 | 485592 | 647456 | 809320 | 971184 |
| t4 (*S. caucasica* split) | 289061 | 273448 | 142317 | 441861 | 7.68E+09 | 546896 | 820344 | 1093792 | 1367240 | 1640688 |
| t5 (*S. magnifica* and *S. caucasica* split) | 345719 | 355526 | 144683 | 490942 | 1.03E+10 | 711052 | 1066578 | 1422104 | 1777630 | 2133156 |
| r1 (backcross) | 0.586416 | 0.61 | 0.276971 | 0.803823 | 0.02104 |  |  |  |  |  |
| r2 (hybridization) | 0.324269 | 0.32 | 0.0335959 | 0.715042 | 0.03273 |  |  |  |  |  |
| N1 | 11398.8 | 11420.5 | 7197.72 | 15276.1 | 6.10E+06 |  |  |  |  |  |
| N2 | 45066.9 | 45161.1 | 33052.6 | 59066.3 | 6.58E+07 |  |  |  |  |  |
| N3 | 59732.2 | 64257.2 | 15996.2 | 96218.6 | 6.77E+08 |  |  |  |  |  |
| N4 | 23124.1 | 22827.2 | 15065.6 | 29462.3 | 1.30E+07 |  |  |  |  |  |
| N5 | 53716 | 54972.7 | 6469 | 95687.8 | 7.13E+08 |  |  |  |  |  |
| N6 | 51202.5 | 49294.4 | 6088.5 | 96941.6 | 7.85E+08 |  |  |  |  |  |
| NA | 61933.4 | 68427.8 | 6879.63 | 97555.1 | 8.05E+08 |  |  |  |  |  |
| Nc | 54195.7 | 54822 | 7527.86 | 97215.5 | 8.29E+08 |  |  |  |  |  |
