# Supplementary material for: Hybridization and introgression events in cooccurring populations of closely related grasses (Poaceae: Stipa) in high mountain steppes of Central Asia
Source: PLoS One. 2024 Feb 27;19(2):e0298760. doi: 10.1371/journal.pone.0298760 (PMC10898772; doi:10.1371/journal.pone.0298760)
Supplement: S8 Table — Note: N is the number of estimated population size, t is time (generations) and set within the range 10–500000, r is population admixture rate. (DOCX) [file pone.0298760.s008.docx]

**S8 Table**. **Parameter estimation for chosen scenario for *S. lingua*, *S. caucasica* and their putative hybrid based on DIYABC.** Note: N is the number of estimated population size, t is time (generations) and set within the range 10–500000, r is population admixture rate.

|  |  |  |  |  |  | **Years (time)** | | | | |
| --- | --- | --- | --- | --- | --- | --- | --- | --- | --- | --- |
| **Parameter** | **Expectation** | **Median** | **Quantile_0.05** | **Quantile_0.95** | **Variance** | **2** | **3** | **4** | **5** | **6** |
| t1 (hybridization) | 9489.69 | 6132.97 | 637 | 25779.9 | 2.03E+07 | 12265.94 | 18398.91 | 24531.88 | 30664.85 | 36797.82 |
| t2 (*S. caucasica* split) | 250152 | 247734 | 96913.2 | 390524 | 7.29E+09 | 495468 | 743202 | 990936 | 1238670 | 1486404 |
| t3 (*S. lingua* and *S. caucasica* split) | 352392 | 360715 | 206454 | 483142 | 9.49E+09 | 721430 | 1082145 | 1442860 | 1803575 | 2164290 |
| r1 (hybridization) | 0.448546 | 0.45 | 0.396742 | 0.50308 | 0.00016 |  |  |  |  |  |
| N1 | 10084.1 | 9863.77 | 4506.55 | 14186.5 | 2.66E+07 |  |  |  |  |  |
| N2 | 31917.6 | 31045.2 | 15406.2 | 48483.5 | 1.26E+08 |  |  |  |  |  |
| N3 | 67673.5 | 69929.7 | 21186.7 | 98245.5 | 5.65E+08 |  |  |  |  |  |
| N4 | 26022.9 | 25465.4 | 17591.5 | 35591.4 | 2.78E+07 |  |  |  |  |  |
| NA | 54283.1 | 55380.8 | 3313.64 | 97911.5 | 9.86E+08 |  |  |  |  |  |
| Nc | 60679.4 | 64130.6 | 11709.1 | 95806.8 | 5.96E+08 |  |  |  |  |  |
