# Supplementary material for: Hybridization and introgression events in cooccurring populations of closely related grasses (Poaceae: Stipa) in high mountain steppes of Central Asia
Source: PLoS One. 2024 Feb 27;19(2):e0298760. doi: 10.1371/journal.pone.0298760 (PMC10898772; doi:10.1371/journal.pone.0298760)
Supplement: S1 Fig — Characters were measured in millimeters (mm). (PDF) [file pone.0298760.s009.pdf]

**S1 Fig. Box plots of 18 quantitative characters for *S. caucasica* subsp. *caucasica*, *S. caucasica* subsp. *nikolai*, *S. magnifica*, *S. lingua*, *S. magnifica* × *S. caucasica*, *S. lingua* × *S. caucasica*.** Characters were measured in millimeters (mm).

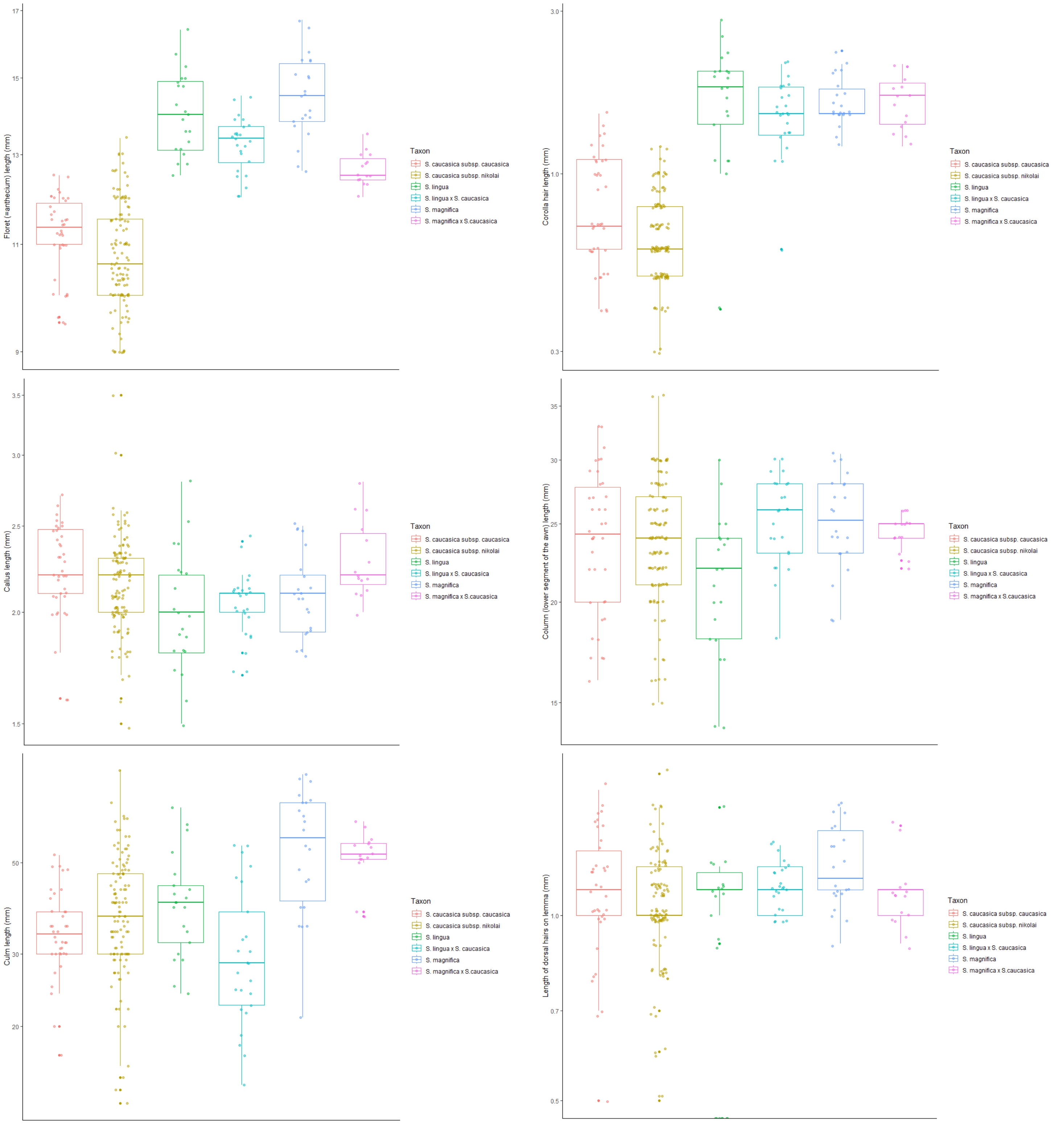

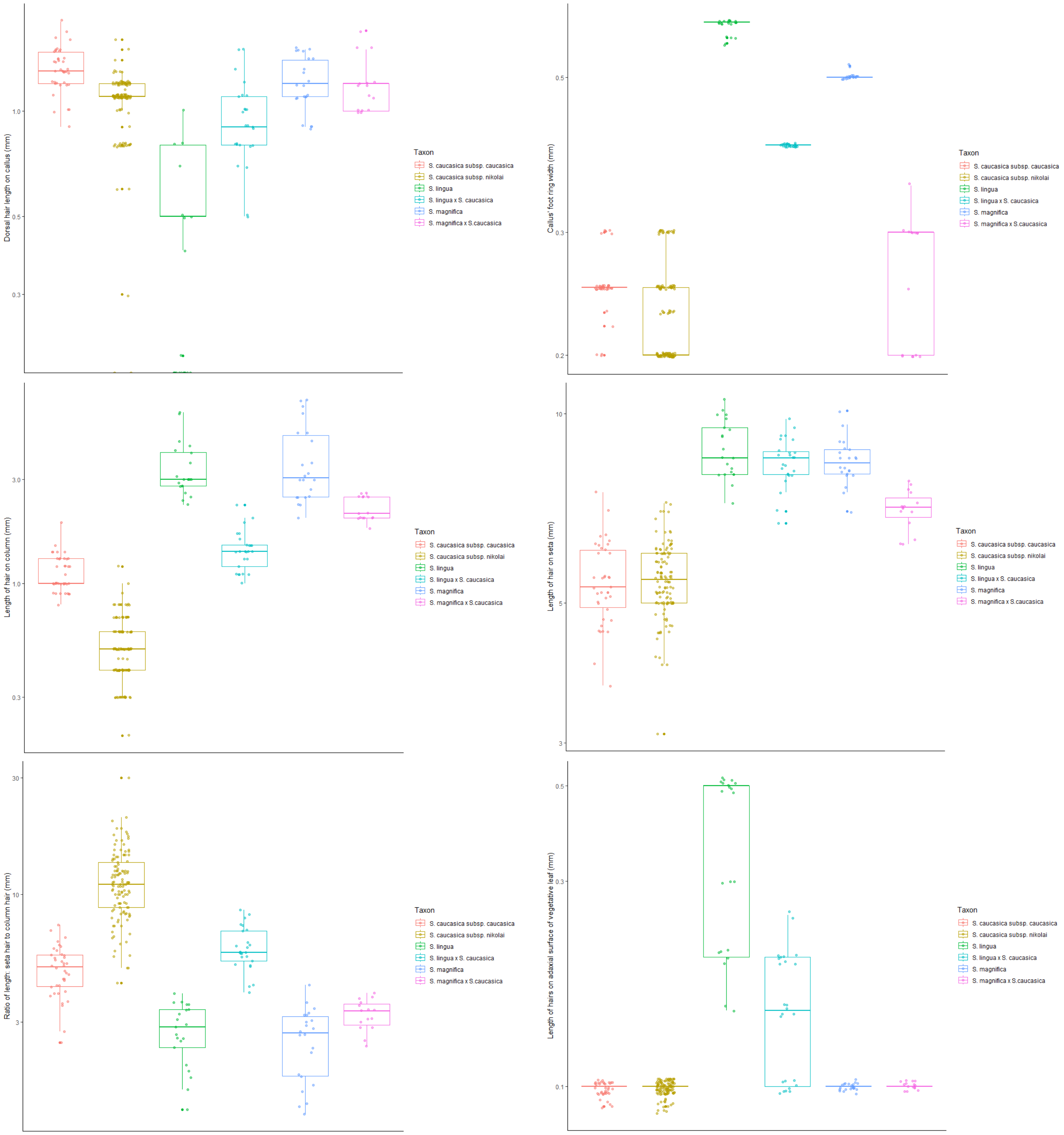
Dorsal hair length on callus (mm)
1.0
0.5
0.3
Taxon
S. caucasica subsp. caucasica
S. caucasica subsp. nikolai
S. lingua
S. lingua x S. caucasica
S. magnifica
S. magnifica x S.caucasica
Callus' foot ring width (mm)
0.5
0.3
0.2
Taxon
S. caucasica subsp. caucasica
S. caucasica subsp. nikolai
S. lingua
S. lingua x S. caucasica
S. magnifica
S. magnifica x S.caucasica
Length of hair on column (mm)
3.0
1.0
0.3
Taxon
S. caucasica subsp. caucasica
S. caucasica subsp. nikolai
S. lingua
S. lingua x S. caucasica
S. magnifica
S. magnifica x S.caucasica
Length of hair on seta (mm)
10
5
3
Taxon
S. caucasica subsp. caucasica
S. caucasica subsp. nikolai
S. lingua
S. lingua x S. caucasica
S. magnifica
S. magnifica x S.caucasica
Ratio of length: seta hair to column hair (mm)
30
10
3
Taxon
S. caucasica subsp. caucasica
S. caucasica subsp. nikolai
S. lingua
S. lingua x S. caucasica
S. magnifica
S. magnifica x S.caucasica
Length of hairs on adaxial surface of vegetative leaf (mm)
0.5
0.3
0.1
Taxon
S. caucasica subsp. caucasica
S. caucasica subsp. nikolai
S. lingua
S. lingua x S. caucasica
S. magnifica
S. magnifica x S.caucasica

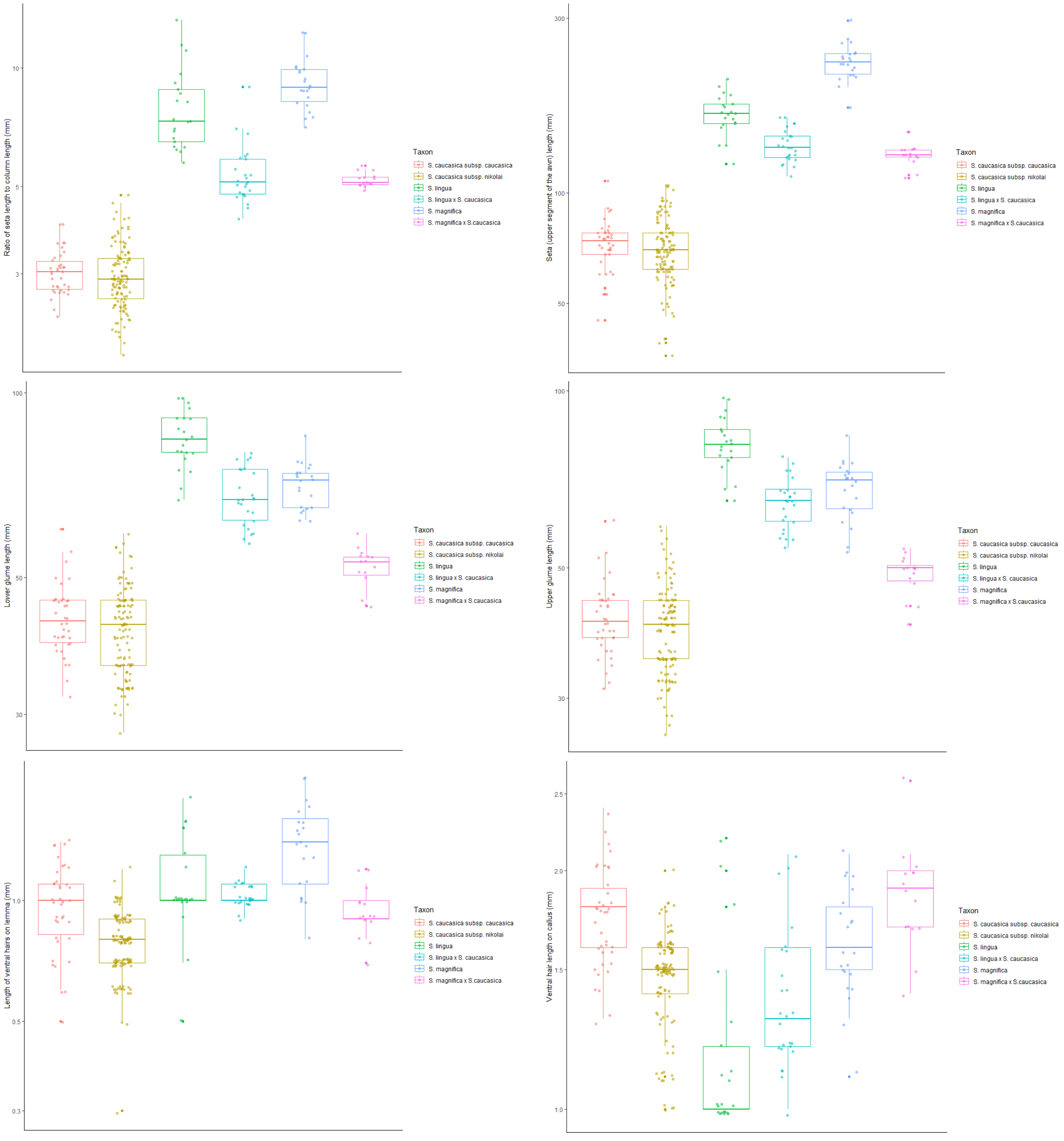
Ratio of seta length to column length (mm)
3
5
10
Taxon
S. caucasica subsp. caucasica
S. caucasica subsp. nikolai
S. lingua
S. lingua x S. caucasica
S. magnifica
S. magnifica x S.caucasica
Seta (upper segment of the awn) length (mm)
50
100
300
Taxon
S. caucasica subsp. caucasica
S. caucasica subsp. nikolai
S. lingua
S. lingua x S. caucasica
S. magnifica
S. magnifica x S.caucasica
Lower glume length (mm)
30
50
100
Taxon
S. caucasica subsp. caucasica
S. caucasica subsp. nikolai
S. lingua
S. lingua x S. caucasica
S. magnifica
S. magnifica x S.caucasica
Upper glume length (mm)
30
50
100
Taxon
S. caucasica subsp. caucasica
S. caucasica subsp. nikolai
S. lingua
S. lingua x S. caucasica
S. magnifica
S. magnifica x S.caucasica
Length of ventral hairs on lemma (mm)
0.3
0.5
1.0
Taxon
S. caucasica subsp. caucasica
S. caucasica subsp. nikolai
S. lingua
S. lingua x S. caucasica
S. magnifica
S. magnifica x S.caucasica
Ventral hair length on callus (mm)
1.0
1.5
2.0
2.5
Taxon
S. caucasica subsp. caucasica
S. caucasica subsp. nikolai
S. lingua
S. lingua x S. caucasica
S. magnifica
S. magnifica x S.caucasica
