# Supplementary material for: Hybridization and introgression events in cooccurring populations of closely related grasses (Poaceae: Stipa) in high mountain steppes of Central Asia
Source: PLoS One. 2024 Feb 27;19(2):e0298760. doi: 10.1371/journal.pone.0298760 (PMC10898772; doi:10.1371/journal.pone.0298760)

S3 Fig. Linear Discriminant Analysis plot of scenario 3 and 4 of the occurrence of *S. magnifica* × *S. caucasica*. These two scenarios are involving hybridization of *S. magnifica* and *S. caucasica* subsp. *nikolai* and backcross to *S. magnifica*.

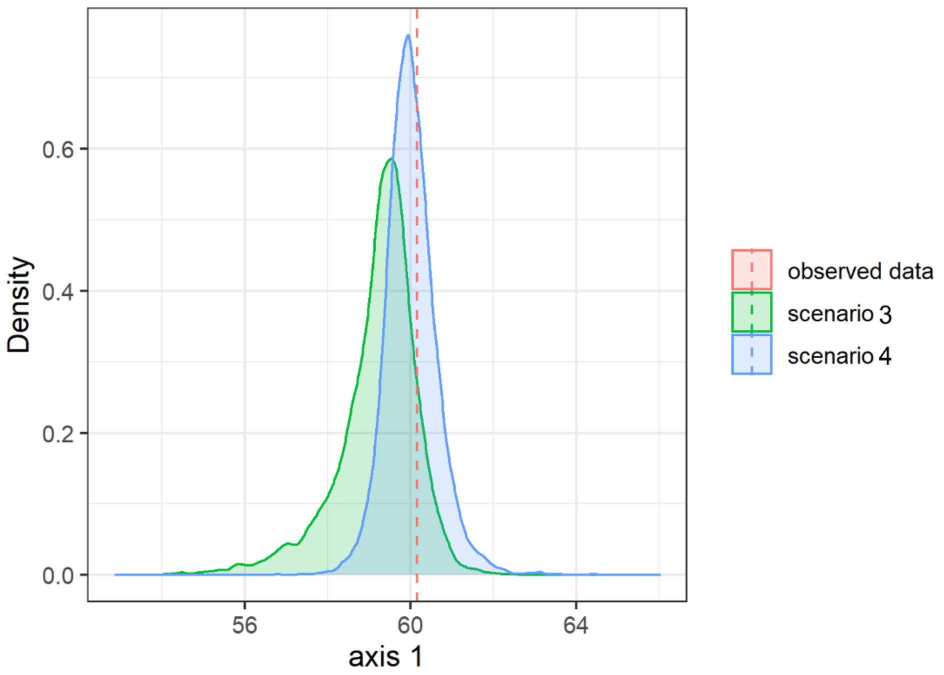

Supplement: S3 Fig — These two scenarios are involving hybridization of S. magnifica and S. caucasica subsp. nikolai and backcross to S. magnifica. (PDF) [file pone.0298760.s011.pdf]
