# Supplementary material for: Hybridization and introgression events in cooccurring populations of closely related grasses (Poaceae: Stipa) in high mountain steppes of Central Asia
Source: PLoS One. 2024 Feb 27;19(2):e0298760. doi: 10.1371/journal.pone.0298760 (PMC10898772; doi:10.1371/journal.pone.0298760)

S4 Fig. Linear Discriminant Analysis plot of scenario 5 and 6 of the occurrence of *S. lingua* × *S. caucasica*. These two scenarios are involving only hybridization of *S. lingua* with *S. caucasica* subsp. *nikolai* or *S. lingua* with *S. caucasica* subsp. *caucasica*.

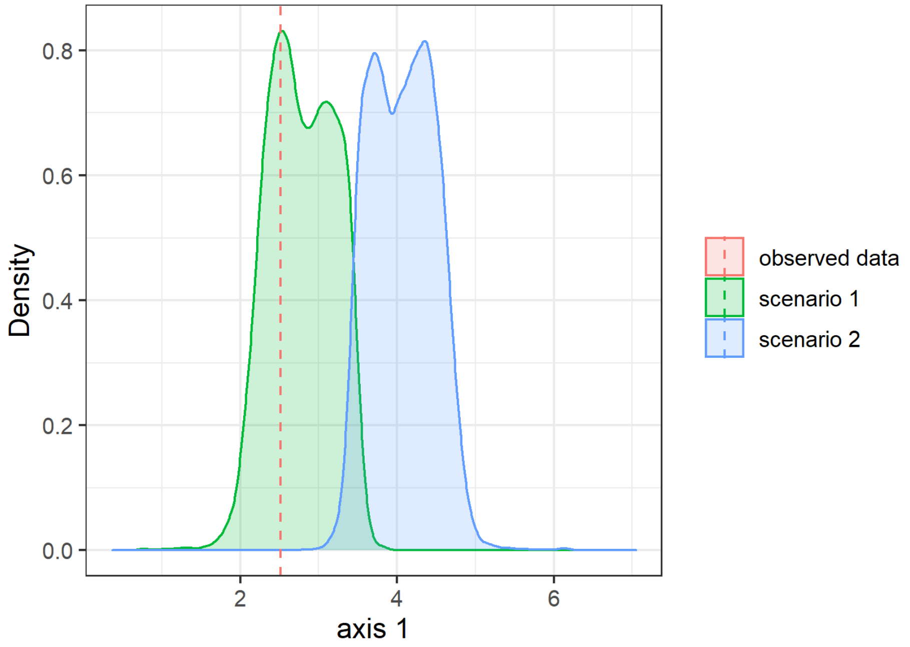

Supplement: S4 Fig — These two scenarios are involving only hybridization of S. lingua with S. caucasica subsp. nikolai or S. lingua with S. caucasica subsp. caucasica. (PDF) [file pone.0298760.s012.pdf]
