# Supplementary figures and images for: Hybridization and introgression events in cooccurring populations of closely related grasses (Poaceae: Stipa) in high mountain steppes of Central Asia
Source: PLoS One. 2024 Feb 27;19(2):e0298760. doi: 10.1371/journal.pone.0298760 (PMC10898772; doi:10.1371/journal.pone.0298760)

**S5 Fig. Holotype of *Stipa* × *muksuensis* M. Nobis, Klichowska, A. Nowak, P. Sinaga.**

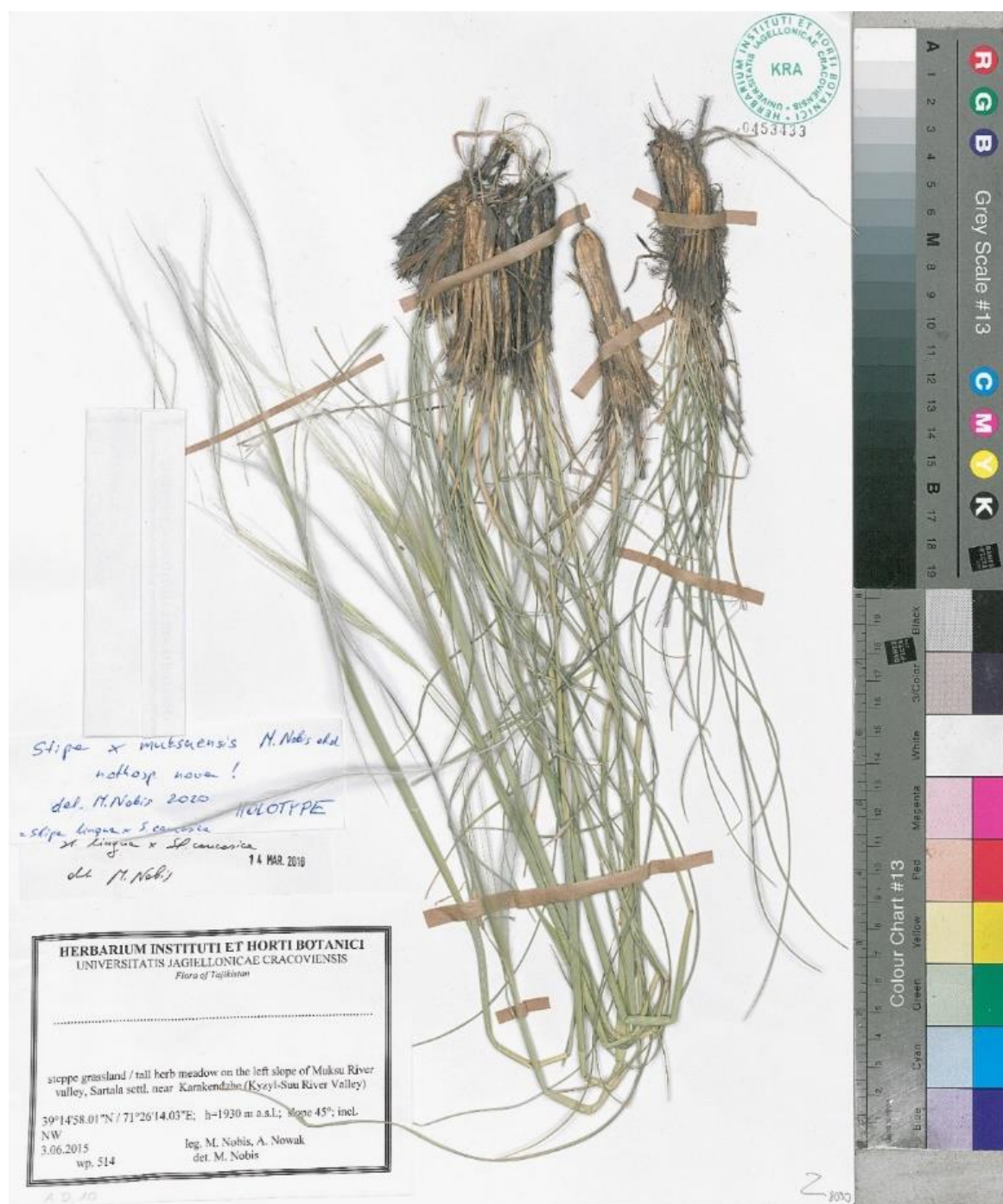

Supplement: S5 Fig — (PDF) [file pone.0298760.s013.pdf]

**S6 Fig. Holotype of *Stipa* × *ochyrae* M. Nobis, Klichowska.**

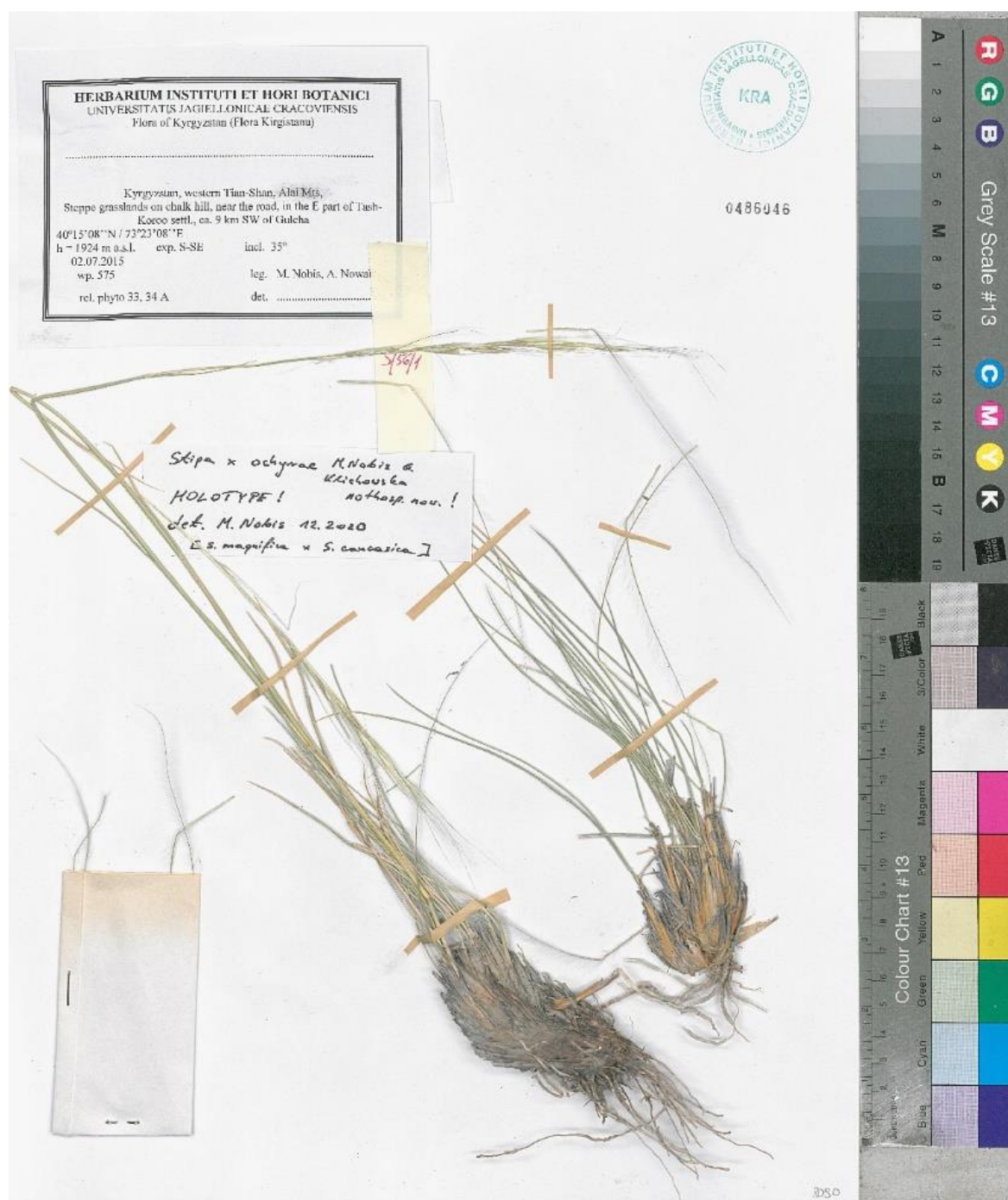

Supplement: S6 Fig — (PDF) [file pone.0298760.s014.pdf]
